# Learning a Weighted Sequence Model of the Nucleosome Core and Linker Yields More Accurate Predictions in *Saccharomyces cerevisiae* and *Homo sapiens*

**DOI:** 10.1371/journal.pcbi.1000834

**Published:** 2010-07-08

**Authors:** Sheila M. Reynolds, Jeff A. Bilmes, William Stafford Noble

**Affiliations:** 1 Department of Electrical Engineering, University of Washington, Seattle, Washington, United States of America; 2 Department of Computer Science and Engineering, University of Washington, Seattle, Washington, United States of America; 3 Department of Genome Sciences, University of Washington, Seattle, Washington, United States of America; Duke University, United States of America

## Abstract

DNA in eukaryotes is packaged into a chromatin complex, the most basic element of which is the nucleosome. The precise positioning of the nucleosome cores allows for selective access to the DNA, and the mechanisms that control this positioning are important pieces of the gene expression puzzle. We describe a large-scale nucleosome pattern that jointly characterizes the nucleosome core and the adjacent linkers and is predominantly characterized by long-range oscillations in the mono, di- and tri-nucleotide content of the DNA sequence, and we show that this pattern can be used to predict nucleosome positions in both *Homo sapiens* and *Saccharomyces cerevisiae* more accurately than previously published methods. Surprisingly, in both *H. sapiens* and *S. cerevisiae*, the most informative individual features are the mono-nucleotide patterns, although the inclusion of di- and tri-nucleotide features results in improved performance. Our approach combines a much longer pattern than has been previously used to predict nucleosome positioning from sequence—301 base pairs, centered at the position to be scored—with a novel discriminative classification approach that selectively weights the contributions from each of the input features. The resulting scores are relatively insensitive to local AT-content and can be used to accurately discriminate putative dyad positions from adjacent linker regions without requiring an additional dynamic programming step and without the attendant edge effects and assumptions about linker length modeling and overall nucleosome density. Our approach produces the best dyad-linker classification results published to date in *H. sapiens*, and outperforms two recently published models on a large set of *S. cerevisiae* nucleosome positions. Our results suggest that in both genomes, a comparable and relatively small fraction of nucleosomes are well-positioned and that these positions are predictable based on sequence alone. We believe that the bulk of the remaining nucleosomes follow a statistical positioning model.

## Author Summary

DNA in eukaryotes is packaged into a chromatin complex, the most basic element of which is the nucleosome. The precise positioning of the nucleosome cores allows for selective access to the DNA, and the mechanisms that control this positioning are important pieces of the gene expression puzzle. In this work, we describe a large-scale DNA sequence pattern that jointly characterizes the sequence preferences of the nucleosome core and the adjacent linkers. We show that this pattern can be used to predict nucleosome positions in both *H. sapiens* and *S. cerevisiae* more accurately than previously published methods. The model is most accurate in predicting the most stably positioned nucleosomes, and describes a sequence composition pattern that determines a locally optimal dyad (nucleosomal DNA mid-point) position. In contrast to some previous models, this model is not based primarily on excluding poly-A/T sequences, nor does the model prefer 10 bp periodicity. Our results suggest that local sequence composition is one of many factors that direct the positioning of nucleosomes, while dynamic processes such as transcriptional elongation and the actions of chromatin remodeling complexes also play a significant role in the overall chromatin landscape.

## Introduction

DNA in eukaryotes is packaged with histone and other proteins into a chromatin complex. The most basic element of chromatin is the nucleosome, which consists of a core of eight histone proteins around which is wound approximately 147 bp of double-stranded DNA. The precise positioning of the nucleosome cores and the inter-nucleosomal linker regions allows for selective access to the DNA by the cellular machinery; understanding the mechanisms that control this positioning is therefore crucial to our understanding of gene regulation and expression.

The recently published high-resolution maps of 20 histone methylations in *H. sapiens* CD4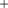 T-cells [1] provided the first *H. sapiens* genome-wide experimental data from which nucleosome positions could be inferred. Barski *et al.* combined chromatin immunoprecipitation (ChIP) with direct high-throughput sequencing of the ChIP DNA samples in the new procedure known as ChIP-seq. To resolve the histone modification signals to individual nucleosomes, templates from purified CD4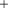 T-cells were created by micrococcal nuclease (MNase) digestion of native chromatin, followed by a mononucleosome-length selection on a gel. The sequencing process resulted in roughly 185 million sequence tags which were unambiguously mapped to the *H. sapiens* genome. Zhang *et al.* developed and applied a computational approach for identifying positioned nucleosomes to this histone-methylation ChIP-seq data, and identified over 438,000 positioned nucleosomes [2]. A subsequent set of experiments by Schones *et al.* eliminated the ChIP step to produce genome-wide maps of nucleosome positions in both resting and activated *H. sapiens* CD4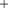 T-cells [3]. These two experiments respectively resulted in 154 million and 142 million unambiguously mapped sequence tags. A similar genome-wide experiment, conducted in *S. cerevisiae* by Field *et al.*, produced 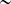
380,000 fully sequenced nucleosomes which were mapped to the *S. cerevisiae* genome with at least 95% identity [4].

In the past three years, at least eight significant papers have described nucleosome positioning models based on DNA sequence signals. A commonly cited nucleosome affinity feature is a 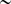
10 bp periodicity of certain dinucleotides, which was first described by Trifonov *et al.* in 1980 [5] and has since been confirmed in both synthetic [6], [7] and natural sequences from a variety of organisms including chicken [8], mouse [7], *S. cerevisiae* [4], [9], [10], worm [10], [11], and *H. sapiens* [12]. The periodic repetition of these sequence elements, with a period that matches the pitch of the DNA helix, is thought to encourage the large-scale bending of the DNA molecule necessary to form a nucleosome. As a result, several computational models have emphasized the presence of this dinucleotide periodicity within the nucleosome core [4], [9], [10], [13]. However, based on a large dataset of *S. cerevisiae* nucleosomes, Mavrich *et al.* [14] observed that an enrichment of AA dinucleotides toward the 5′ end of the nucleosome was in fact a better descriptor of nucleosome positioning than the 10 bp periodicities of AA/TT. In contrast to the computational models derived from short sequences chosen for their high affinity to wrap around histones and form nucleosomes, models derived from larger nucleosome-occupancy datasets have frequently found that the strongest sequence signals are nucleosome-inhibiting rather than nucleosome-forming [4], [14], [15]. Discriminative models [16], [17] as well as regression-based models [15], [18] found that the most statistically significant features were more often *exclusion* signals rather than *occupancy* signals. In the approach described by Peckham *et al.* [16], of the top 17 features only 5 are nucleosome occupancy signals. The same trend was observed by Yuan *et al.* [15] even though their statistical model explicitly sought to extract dinucleotide periodicities using wavelet analysis: out of the 17 selected features only 3 are positive for nucleosome occupancy, and none of the positive features were related to the 10 bp periodicity of any dinucleotide. Lee *et al.* [18] also concluded that nucleosome occupancy is probably more often directed by exclusion signals, and their Lasso-based model assigned the greatest significance to DNA structural features (*e.g.* tilt and propeller twist).

In this work we present a new approach to predicting nucleosome positioning directly from DNA sequence. Although our model also includes features describing dinucleotide and trinucleotide sequence patterns, it was originally inspired by our observation of a dramatic mono-nucleotide sequence pattern surrounding the nucleosome positions identified using the Nucleosome Positioning from Sequencing (NPS) algorithm [2] applied to the Barski *at al.* dataset. We subsequently obtained a nearly identical nucleosome pattern from the Schones dataset derived from resting *H. sapiens* CD4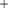 T-cells [3] by using a version of the NPS software that we modified to estimate nucleosome dyad positions rather than nucleosome occupancy regions. An analysis of the distribution of start-to-start and start-to-end distances for the short-read sequencing tags (as described in [11], and shown in Figure S1) indicates that the Schones dataset has more consistent nucleosome-sized start-to-end distances than a combination of the 21 separate ChIP-seq experiments in the Barski dataset. We conclude that nucleosome dyad positions inferred from the Schones dataset have a smaller average error than those inferred from the Barski dataset, and therefore use the Schones data to evaluate the performance of our model.

We show that in both *H. sapiens* and *S. cerevisiae*, the most informative individual features are the mono-nucleotide patterns, although the additional information provided by di- and tri-nucleotide features improves the performance of our sequence scoring method. Our method for computing the dyad score of a given DNA sequence position consists of two steps: first a set of patterns are correlated with the local DNA sequence, and second the resulting correlation values are weighted and summed to produce the final score. The two elements most responsible for our method's discriminative power are the length of the patterns used and the discriminative weights applied to the sequence features. We determined that the optimum pattern length is between 300 and 350 nucleotides—indicating that the DNA sequence pattern of a nucleosome includes not just the core region that is tightly wound around the histone proteins, but the adjacent linkers as well. Notably, this result was the same for both *H. sapiens* and *S. cerevisiae*. In the second step, the weights allow our method to selectively assign greater importance to the more informative features—*e.g.*, the trinucleotide AAA is given a higher weight than GTA. By examining the patterns associated with and the classification performance achieved by each of the mono, di- and tri-nucleotides, we may also be able to gain a deeper understanding of the forces that influence nucleosome positions within the chromatin structure, and to what extent these forces are consistent between *H. sapiens* and *S. cerevisiae*. Toward this end, we hypothesize that the close proximity of the two superhelical coils within each nucleosome and the structure of the 30 nm fiber also play a role in determining the DNA sequence preference of nucleosomes.

The dyad scores produced by our method are relatively insensitive to local AT-content and can be used to accurately discriminate dyad positions from adjacent linker regions without requiring an additional dynamic programming step to capture the linker-nucleosome-linker pattern. Although not required, such a post-processing step can be easily applied to these scores in order to estimate the probability that a nucleosome is centered at any particular genomic position or that a particular nucleotide is “occupied” by a nucleosome, as has been done previously [4], [19]. While such a post-processing step entails making assumptions regarding overall nucleosome density and the distribution of linker lengths, it can be used to find the most likely parse of a DNA sequence into nucleosomes and linkers, and to compute posterior probabilities of nucleosome occupancy at each position along the sequence. The most likely parse identifies nucleosome positions which can then be compared to experimentally estimated nucleosome positions, an evaluation method which has been used in, *e.g.* [9], [16]. Posterior probabilities of nucleosome occupancy provide normalized scores which are more amenable to computing average landscapes of nucleosome occupancy surrounding genomic features such as transcription start sites. In this work we choose to evaluate our dyad-scoring method by testing how well the raw scores are able to discriminate dyad positions from adjacent linker regions, a similar but more stringent evaluation criterion than has been used previously [4], [10], [16], [17].

We present an evaluation of our method on the Schones dataset derived from *H. sapiens* T-cells, as well as on the genome-wide *S. cerevisiae* data made available by Field *et al.* [4]. In addition, we compare our approach to two recently published methods [4], [10] and show that our method is significantly better at discriminating dyad positions from adjacent linkers. Further, we compare the *H. sapiens* trained patterns to the *S. cerevisiae* trained patterns and find large-scale similarities despite the presence of 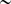
10 bp periodicities in the *S. cerevisiae* patterns and the striking lack thereof in the *H. sapiens* patterns. We also apply our method to the entire *H. sapiens* and *S. cerevisiae* genomes as well as to specific subsets of interest including transcription start sites, CTCF binding sites, and *H. sapiens* repetitive elements.

## Results

### Highly significant pattern of mono-nucleotide oscillations found in *H. sapiens* nucleosomal sequences

**Figure 1:**
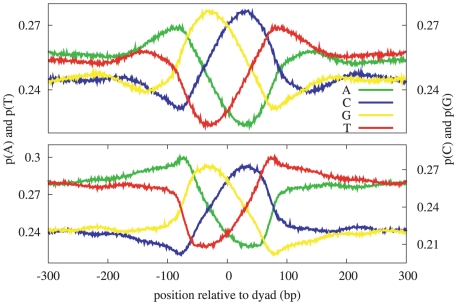
Mono-nucleotide patterns in *H. sapiens*. These patterns were derived by aligning DNA sequences at experimentally determined nucleosome dyads, and computing the resulting position specific frequency matrix. The correlation between the corresponding mono-nucleotide patterns derived from the Barski nucleosome positions (top) and the Schones nucleosome positions (bottom) is 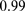.

A recently published dataset [2] provides the largest collection to date of experimentally determined *H. sapiens* nucleosome positions. This set of 438,652 nucleosome positions, which we will refer to as the Zhang positions, was derived from the histone methylation ChIP-seq data from CD4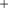 T-cells [1] using the NPS algorithm [2]. Each nucleosome position in the Zhang dataset is a short segment of DNA, specified by a pair of chromosome coordinates, and is annotated with a p-value and a list of histone marks. Estimating the nucleosome dyad position as the mid-point of each of the Zhang nucleosome regions, we extracted DNA sequence from the reference genome centered at each of these dyad positions, and we computed the mono-nucleotide position specific frequency matrix shown in Figure 1 (top). Far from the nucleosome dyad (as shown in Figure S2), the background GC fraction is 0.46, which is higher than the *H. sapiens* genome-wide average of 0.41, and consistent with the known bias of the Barski *et al.* dataset toward GC-rich regions of the *H. sapiens* genome. In the nucleosome core, however, the average GC content is significantly higher than the average AT content. Within a narrow window around the dyad, a nucleosome-sized pattern is observed. The pattern is its own reverse-complement: the A and T traces mirror each other across the dyad, as do the C and G traces, and the pattern emerges even when only the reference strand is used for each nucleosome positions. (Using both strands enforces this reverse-complement symmetry by construction.) The reverse-complement symmetry is an expected consequence of the dyad symmetry of the nucleosome particle. However, the fact that each trace is not itself symmetrical around the dyad axis is intriguing and shows that there is a directionality to the nucleosome which obeys the antiparallel, complementary nature of the double-stranded helix: the highest local density of G's and the lowest local density of T's occur 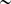
40 nucleotides 5′ of the dyad, and the highest local density of C's and the lowest local density of A's occur 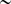
40 nucleotides 3′ of the dyad.

**Figure 2:**
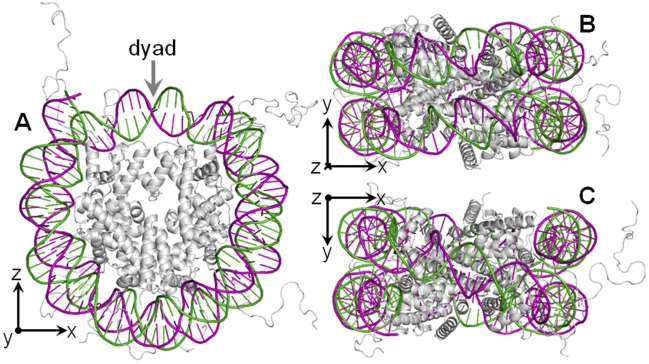
X-ray structure of the nucleosome core particle. These views of NCP147, at 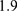
Å resolution, show the two strands of the double-helix in purple and green, with the protein core in grey. (A) shows the curvature of DNA around the histone core, with the dyad at the top, center; (B) represents a 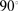 rotation of the particle, showing the adjacent segments of DNA, opposite the dyad; and (C) represents a 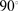 rotation in the opposite direction, showing the DNA crossing over the dyad. As indicated by the coordinate system axes, in (A) the y-axis is pointing out of the page, in (B) the z-axis is pointing into the page, and in (C) the z-axis is pointing out of the page.

The dominant hypothesis regarding DNA sequence preference of nucleosome formation is related to the curvature required to wrap the double helix tightly around the histone core [20]. However, as illustrated in Figure 2a, the curvature is relatively uniform throughout the nucleosome core [21] and therefore, while this hypothesis explains the frequently observed 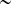
10 bp periodicity, it does not explain asymmetric patterns such as those shown in Figure 1, with extrema at some distance from the dyad. We propose that two other structural aspects of the chromatin may explain why the extremes of the nucleosome pattern (local maxima for C and G, and local minima for A and T) are centered approximately 40 bp on either side of the dyad rather than at the dyad itself. The first structural aspect that we will consider is the close proximity of the two superhelical coils within each nucleosome: DNA regions that are 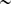
80 bp apart are brought into close proximity [22], as shown in Figure 2b, while the 10–20 bp immediately surrounding the dyad are not in similarly close proximity to another double helix, as shown in Figure 2c. Specifically, base pair 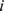 is brought into close proximity with basepair 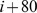, for 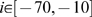 (with the dyad defined as position 0). If this close proximity of the two double-helices has an effect on the nucleosome sequence preference, this effect would be observed most strongly 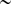
40 bp on either side of the dyad. The second structural aspect is related to the structure of the “30 nm fiber”. Although this structure is not yet well understood, all proposed structures are such that dyads face the center of the fiber while the DNA regions 20–60 bp on either side of the dyad form the exterior of the fiber [23]–[25]. We hypothesize that DNA regions on the outside of the 30 nm fiber may experience different selective pressures than regions on the inside of the fiber, and the result of this difference would be a nucleosome sequence pattern with extreme deviations centered approximately 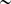
40 bp on either side of the dyad. We note that while both of these hypotheses are consistent with the asymmetric patterns presented here, they would also be consistent with symmetric, M- or W-shaped patterns with local maxima or minima at 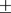
40 bp.

In order to quantify the significance of the pattern shown in Figure 1 (top), we consider each of the four mono-nucleotide traces separately. The most striking aspect of the pattern is the relatively large variation in the probability of each of the nucleotides, across a distance of less than 150 bp, averaged over more than 400,000 DNA segments. Based on a null model of a similarly constructed pattern using randomly sampled DNA segments, we estimate the probability that this observed variation could occur by chance to be 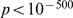. (See Methods for details and Figure S3 for a plot of the null model distribution.)

In order to determine whether the observed pattern might be the result of an artifact in part of the dataset, we considered the possible impacts of varying AT-content and repetitive sequences. We found that each of the five patterns obtained after partitioning the data into quintiles according to AT-content were similar to the original pattern, disregarding vertical translations of the individual components reflecting increases in AT content and corresponding decreases in GC content (Figure S4). Partitioning the dataset into three subsets according to the distance to the nearest repeat also does not significantly alter the shape of the pattern (Figure S5).

We noted earlier that the A and T traces mirror each other across the dyad, as do the C and G traces, and that intriguingly A and C mirror each other across a horizontal line of symmetry, as do the T and G traces. The first symmetry, of A/T and C/G across the dyad, is a natural consequence of the dyad symmetry of the nucleosome, while the second A/C and T/G symmetry is not. Although a similar downward trend 5′ to 3′ across the nucleosome dyad and a local minimum 3′ of the dyad in the AA dinucleotide frequency can be seen in the figures in an early paper by Ioshikhes *et al.* [26], the trend was not explicitly noted. Instead the authors emphasized the asymmetry in the peaks of the dinucleotide patterns and found that the 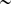
10 bp periodicity exhibited by the AA and TT dinucleotides had opposite phase, in contrast to the same-phase periodic pattern described earlier by Satchwell *et al.* [8] and more recently by Segal *et al.* [9]. More recently, the enrichment of AA dinucleotides 5′ of the dyad and TT dinucleotides 3′ of the dyad has been described [14], although no biological hypothesis for this directional preference has yet been suggested.

We will refer to the pattern illustrated in Figure 1 as AGCT, based on the 5′ to 3′ ordering of the local maxima. This simple pattern is consistent with the common nucleosome model: higher AT content in the linker regions and higher GC content in the core. We hypothesized the existence of alternative forms of this pattern in which the ordering of the individual nucleotides is permuted while conforming to the common model—the other possible patterns would be ACGT, TGCA, and TCGA. To test this hypothesis, we created models of all four pattern variants and partitioned the input set of sequences according to which of the four patterns best matched each individual sequence (if a particular DNA sequence did not correlate well with any pattern, it was assigned to the no-match partition). We found, as expected, that more of the input sequences correlated well with the AGCT pattern than with any other pattern (25%). However, our hypothesis was validated in that an even larger fraction (32%) of the sequences correlated well with one of the other three patterns (details in Supplement and Figure S6).

### Identical pattern derived from independent *H. sapiens* dataset

To verify that this nucleosome pattern is not an artifact of the Barski *et al.* dataset, we estimated nucleosome dyad positions from the tag coordinate files for resting CD4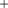 T-cells published by Schones *et al.* [3]. The experimental procedure used by Schones *et al.* is very similar to the one used by Barski *et al.*, but without the ChIP step used to isolate specific histone modifications. We modified the NPS software [2] so that it would output nucleosome dyad positions rather than variable length nucleosome regions (see Methods), and applied it to the Schones dataset. The result was a list of over 828,000 nucleosome dyad positions with NPS-assigned p-values 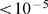. The mono-nucleotide patterns learned from this independently derived list of nucleosome positions are nearly identical to the corresponding patterns derived from the Zhang positions (Pearson correlation 0.99), as shown in Figure 1. In addition, we computed patterns based on the top-50% and top-25% scoring dyad positions (corresponding to p-value thresholds of 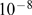 and 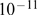 respectively), and found the correlations between the patterns derived from the full set and these subsets to also exceed 0.99, indicating that the pattern is stable and can be learned from smaller datasets. A subset of the dinucleotide patterns are shown in Figure S7. The patterns for AT, TA, GC, and CG are symmetric about the dyad, as expected, because each dinucleotide is its own reverse complement. What is intriguing, however, is that the standardized pattern for CG is nearly identical to that for GC—despite the dramatically different occurrence rates of these two dinucleotides. The standardized patterns for TA and AT are also nearly identical.

We note that neither of these independently derived mono-nucleotide patterns show evidence of 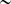
10 bp periodicity, and this is also true of similarly computed dinucleotide patterns. There are two possible explanations for this lack of a periodic component in this pattern. First, the NPS software uses a bin size of 10 nucleotides in processing the short-read sequencing data and estimating the dyad positions, resulting in an average error of at least 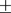
5 nucleotides in each position estimate which would smooth out any 10 bp periodicity in the average pattern. Second, 10 bp periodicity of the AA dinucleotide has to date been observed only in small sets of *H. sapiens* nucleosomal sequences and is not observable on a genome-wide scale in *H. sapiens*, in sharp contrast to *S. cerevisiae* [12], [27]. While removing the binning step in the NPS process may yield more accurate dyad positions, we caution that it may also amplify the impact of the MNase sequence specificity as is apparent in the higher-resolution yeast data set discussed below. Furthermore, 10 bp periodicity has been most apparent in *alignments* of nucleosomal sequences but has not been shown to be a significant factor in identifying and classifying such sequences.

### Pattern in *S. cerevisiae* follows similar trend, with additional 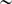
10 bp periodicity component

A recently published genome-wide experimental assay in *S. cerevisiae* produced a dataset of 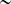
380,000 fully-sequenced nucleosomal sequences [4]. This experiment was based on a sequencing technology capable of 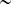
200 bp reads, thereby eliminating the uncertainty inherent in the Barski and Schones datasets regarding the precise lengths of the MNase-cleaved DNA fragments. Estimating dyad positions from the genomic positions of these nucleosomal sequences can therefore be done using a simpler approach (see Methods), which produced a total of 50,815 unique dyad positions. This is a significant fraction of the estimated 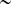
70,000 nucleosomes required by the entire 12 Mb genome. We divided this set of dyad positions according to the confidence associated with each position (estimated as the number of locally overlapping reads), to produce successively smaller subsets of size 25384, 12698, 6355, and 3180 respectively, with each dyad position in the smallest subset estimated from an average of 20 overlapping reads.

**Figure 3:**
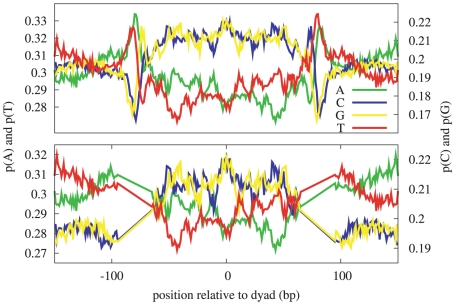
Mono-nucleotide patterns in *S. cerevisiae* with MNase sequence-specificity artifact. These patterns were derived from 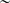
25,000 sequences aligned at experimentally determined dyad positions. The top figure illustrates the MNase sequence specificity artifact at a distance of 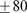 bp from the dyad. To remove this artifact, we linearly interpolated across a 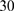 bp region as shown in the bottom figure. (The vertical axis scales are different in the two figures.)

**Figure 4:**
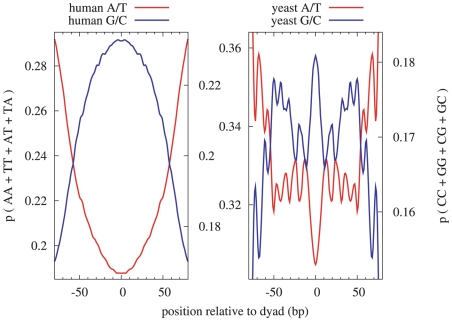
Dinucleotide A/T and G/C patterns. These figures show the frequency of dinucleotides composed exclusively of A/T (red) and G/C (blue). The *H. sapiens* patterns show no evidence of 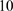 bp periodicity, while the *S. cerevisiae* patterns do, with peaks in the A/T pattern at 13, 24, 36, 47, 58, and 68, and peaks in the C/G pattern at 0, 20, 32, 42, 50, 61, and 72 bp from the dyad. The larger-scale trends of increasing GC-content and decreasing AT-content near the dyad are, however, similar between the two species.

The patterns derived from this set of *S. cerevisiae* dyad positions, as shown in Figure 3, include two elements not present in the *H. sapiens* nucleosome patterns: a very strong artifact due to the MNase sequence bias at a distance of 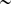
80 bp on either side of the dyad (corresponding to roughly half of the mean read length of 156 nucleotides), and evidence of 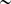
10 bp periodicity for certain dinucleotides. Figure 4 shows the summed patterns for A/T-only and C/G-only dinucleotides, illustrating the lack of apparent periodicity in *H. sapiens* as compared to *S. cerevisiae*, although we also note that the artifact in the *S. cerevisiae* patterns due to the MNase sequence-specificity has a significantly larger amplitude than the 10 bp periodicity. The common elements between *S. cerevisiae* and *H. sapiens* include the downward trend across the dyad of the A nucleotide in the 5′ to 3′ direction, the corresponding upward trend of the T nucleotide, and the local minima in the A- and T-patterns at 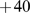 bp and 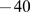 bp respectively. The standardized patterns for *S. cerevisiae* and *H. sapiens* for A, AA, and AAA are shown in Figure S8. Aside from the 10 bp periodicity evident in the *S. cerevisiae* patterns, the overall shapes of these patterns are strikingly similar (Pearson correlations are A:0.77, AA:0.88, AAA:0.91), suggesting that perhaps it is this wider underlying oscillation, more than the 10 bp periodicity, which promotes nucleosome positioning across species.

To avoid biases arising from the MNase sequence specificity, Field *et al.* restricted their model to the 127 positions centered at the dyad [4]. The presence of this artifact in the nucleosome patterns is also an indication that many of the estimated dyad positions are shifted by a few nucleotides from the true positions—more accurate dyad positions could potentially be estimated by inserting an alignment step in the pattern-estimation procedure similar to [26], [28]. We instead eliminate the MNase artifact while still learning a large-scale pattern by linearly interpolating each pattern across a 30 bp width centered at 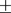
80 as shown in Figure 3.

### Discriminative nucleosome pattern model

Based upon the observed oscillatory pattern of nucleotide composition across the nucleosome, we present here a novel approach to predicting nucleosome positioning from DNA sequence alone. Previous methods have frequently taken a hypothesis-testing approach common in motif-finding algorithms in which a *foreground* or *motif* model score is compared to a *background* model score. In determining the nucleosome-formation potential of a given DNA sequence, a nucleosome model is used to compute a score under the nucleosome hypothesis, and a linker model is used to compute a score under the null (linker) hypothesis. The final score is typically either a ratio or a difference of these two scores. For a given input DNA sequence (the length of which varies depending on the specific implementation but is generally 147 nucleotides or less), the basic question being asked is thus: which of these two models best represents this particular sequence? When scoring the likelihood that a particular nucleotide is at the center of a nucleosome, we have found that using a wider sequence window asks the more appropriate question: do the 147 nucleotides centered at this position fit our model of the nucleosome core *and* do the adjacent regions fit our model of the linker region? In previously published approaches, this alternating linker-nucleosome-linker model is captured by a subsequent dynamic-programming step (*e.g.* [9]), but we will show that the predictive power of the model can be significantly improved by including this longer pattern directly into the initial scoring function.

Based upon our observation that the overall shape of the mono-nucleotide pattern first derived using the Zhang positions was relatively insensitive to local AT-content, our initial insight was that the model should be insensitive to the average local sequence composition. This goal is consistent with the biological requirement for packaging DNA sequences with widely varying AT-content not only within any one genome but across the genomes of all eukaryotes [20]. We avoid inherent sequence composition bias by comparing the input DNA segment to the *shape* of the nucleosome pattern using a Pearson correlation which disregards vertical scaling or translation of the individual pattern components. Further, rather than framing the model in a probabilistic setting, we choose to take the more general approach of extracting an arbitrary number of informative, sequence-related features which are then individually weighted and combined to produce a final dyad score. The complete details of our algorithm are given in the Methods section, but we will outline the basic approach below.

Given an input sequence 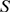, of (odd) length 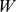, we extract a number of descriptive features and compute the dyad score for the mid-point of sequence 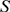 as the weighted sum of these features. The primary features in our model are correlation coefficients: each one represents the correlation between a previously learned pattern 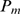 and the new input pattern 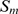 for a given k-mer 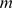, of length 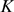. Based on our earlier observation that individual components within the pattern are occasionally reversed, each input pattern 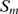 is compared to two versions of the trained pattern: 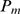 and 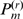 where 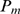 is the *m*-pattern learned from the training set of nucleosome sequences aligned at the inferred dyads, and 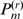 is simply the reflection of 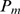 across the axis of symmetry at the dyad. We further add, as secondary features, the number of occurrences of each k-mer 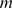 in the input sequence 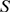 and its reverse-complement. The intuition here is that a correlation coefficient of 0 could be the result of sequence 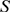 containing zero occurrences of 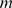, or it could be a true lack of correlation between two non-zero vectors. Likewise, a high correlation score may be more significant if it is based on a sequence with a high number of occurrences of *m*.

In the final step of the training process, we train a binary classifier known as a linear support vector machine (SVM) [29], [30] to discriminate between two sets of examples, each of which is described by a vector of the features defined above and is labeled either *positive* (dyad) or *negative* (linker or non-dyad). The output of this training step is a set of feature-specific weights which, when applied to the set of training examples, optimizes the discrimination between the positive and negative examples. These weights can subsequently be used to compute a score (the dyad score) for any future test example. The sign of the score indicates which side of the decision boundary the test example falls on, and the magnitude of the score is an indication of the confidence of the classification. Although previously published models have commonly used long linkers or nucleosome-free regions as negatives in training and evaluation [4], [15], each nucleosome core is flanked by two linker regions, and we define a more stringent discrimination task by testing how well each dyad position in the test set can be distinguished from the corresponding set of adjacent linkers.

**Figure 5:**
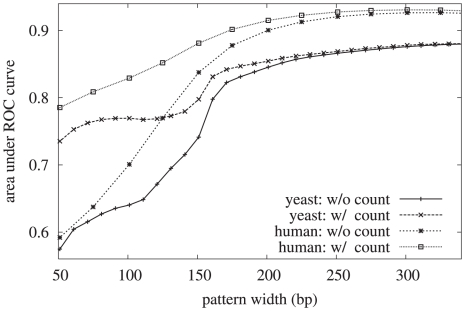
Area under the ROC curve as a function of pattern width. The classification performance was evaluated on one dataset each for *S. cerevisiae* and *H. sapiens*. The impact of the kmer counts feature was also examined and found to be most significant at smaller pattern widths, and not significant for widths beyond 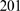 bp.

In order to completely define the model described above, the width of the individual patterns 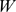, the k-mers of interest, and the distance 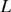 to the linker positions to be used as negative training examples need to be specified. Note that these linker positions and the nucleotide sequence surrounding them are used only in training the SVM weights and do not affect the learning of the k-mer patterns. Further, there is no implicit relationship between the values of 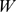 (the pattern width) and 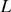 (the distance between the dyad positions and the “negatives” examples). We evaluated the effect of these two parameters on the discrimination performance of our model and found that the width of the patterns (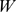) has the most significant effect on the ultimate performance, as shown in Figure 5. For shorter patterns (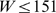) the kmer-count features significantly improve the performance, while for longer patterns (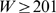) they provide relatively little improvement. The optimum pattern width varies somewhat across different datasets, but is generally between 301 and 351 nucleotides, *i.e.* extending 150–175 positions on either side of the dyad. We also examined the sensitivity of the discrimination performance to the distance 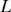 between the dyad and non-dyad positions. When this distance is zero, it is of course impossible to discriminate between the two sets, and the resulting area under the ROC curve is 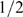. We would expect the performance to improve as the distance increases, up to a maximum value when the distance is roughly half the inter-dyad distance. Beyond this point, we would expect the performance to begin to get worse as the “negative” position approaches the neighboring dyad, and this intuition is borne out by the experimental results shown in Figure S9. The peak performance occurs when the distance between the positive and negative examples is 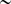
110–120 nucleotides. Based on these analyses, our final model is defined with patterns of width 301 nucleotides, and the SVM is trained with negative examples at a distance of 110 nucleotides on either side of each dyad. We chose to limit our set of k-mers to those of length 1, 2, and 3. Using longer k-mers would require exponentially more parameters, and we found that the improvement gained even by adding trinucleotides was relatively small.

We evaluate our method using a cross-validation approach to ensure that the model is not over-fitting the data. For each chromosome 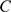, the training set contains all dyad positions 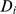 *not* on chromosome 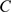, and the held-out test set consists only of those dyad positions on chromosome 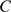. The training of the model consists of three steps: first, a position-specific pattern 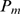 of width 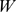 is learned for each k-mer 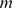 from the sequence centered at each 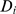 in the training set. Second, features describing the local context of each position 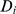 as well as 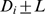 are computed: these include correlation scores against each of the learned patterns and counts for each of the k-mers. Third, these feature vectors and labels are used to train a linear SVM. The evaluation on the held-out test set involves similarly computing features describing the DNA sequences centered at each 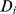 and 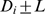 on chromosome 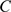, and computing scores for each by using the SVM weights learned during training. The ability of these scores to discriminate between the dyad and non-dyad positions in the test set are evaluated using standard ROC analysis.

### Model classification performance

**Figure 6:**
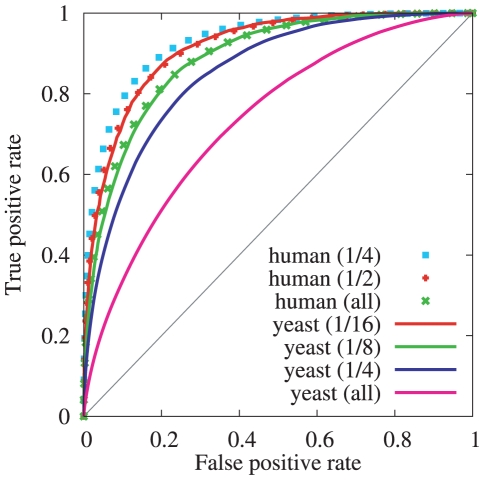
Cross-validated classification performance on *H. sapiens* and *S. cerevisiae* datasets. The *H. sapiens all* dataset contains 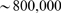 dyad positions, and the *S. cerevisiae all* dataset contains 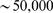 positions. In all cases, the set of negative examples is twice as large as the set of positive examples, and the negative positions are 110 bp away from the dyads. The area under the ROC curves for *H. sapiens* are 0.93, 0.91, and 0.89. The area under the ROC curves from *S. cerevisiae* are 0.91, 0.89, 0.85, and 0.74.

The datasets we used to train and test this model were described earlier and consisted of 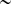
800,000 *H. sapiens* dyad positions estimated from the Schones dataset, and 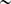
50,000 *S. cerevisiae* dyad positions estimated from the Field dataset. Although different methods were used to create each of these sets of dyad positions, both include experimentally-derived confidence scores. We used these confidence scores to further subdivide each dataset by repeatedly taking the top-scoring half, resulting in 3 *H. sapiens* sets (all, top 1/2, and top 1/4) and 5 *S. cerevisiae* sets (all, top 1/2, top 1/4, top 1/8, and top 1/16). We trained our *H. sapiens* nucleosome model on the top 1/4 subset (approximately 200,000 dyad positions), and we trained the *S. cerevisiae* nucleosome model on the top 1/2 set (approximately 25,000 dyad positions). Figure 6 shows the composite results for our model, based on chromosome-by-chromosome cross-validated training and testing. The number of dyad positions on each chromosome and the per-chromosome area under the ROC curve for each dataset are provided in Tables S1 and S2 in the supplement. The area under the ROC curve is an indication of how well our model discriminates between dyad and linker positions, and the fact that the model performance improves for the highest-scoring subsets shows that, on average, the most-consistently positioned nucleosomes are also the ones given the highest scores by our model, while the adjacent dyads are simultaneously given lower scores. We also found that the difference between the cross-validated results shown here and those obtained when using all of the data for both training and testing were negligible, due to the large amount of training data and the relatively simple model being trained.

We note that the performance for the top 1/4 *H. sapiens* dataset is nearly identical to the performance for the top 1/16 *S. cerevisiae* dataset, and the same is true for the top 1/2 *H. sapiens* dataset and the top 1/8 *S. cerevisiae* dataset. The top 1/16 *S. cerevisiae* dataset contains positions sampled on average every 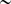
4000 bp across the entire *S. cerevisiae* genome. Assuming an average nucleosome repeat length of 170 bp, this represents approximately 4% of all nucleosomes. The comparable *H. sapiens* dataset, based on the ROC curve, is the top 1/2 set which consists of positions sampled on average every 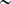
7500 bp across the *H. sapiens* autosomes. (The X and Y chromosomes are significantly undersampled as compared to the autosomes, so we exclude them in this analysis.) Considering the limits imposed by sequencing depth and the unique mapping of short sequence tags to the *H. sapiens* genome, it seems reasonable to suggest that perhaps half of the highly-positioned nucleosomes were missed in the genome-wide Schones experiment. With this assumption we estimate that approximately 4% of the nucleosomes in *both H. sapiens* and *S. cerevisiae* are positioned consistently enough across a population of cells to produce an area under the ROC curve of 0.91, which corresponds in this case to a true positive rate of 73% at a false positive rate of 10%. Doubling the size of the set takes us to the next curve for each species: approximately 8% of nucleosomes are positioned consistently enough to produce an area under the ROC curve of 0.89, which corresponds in this case to a true positive rate of 66% at a false positive rate of 10%.

**Figure 7:**
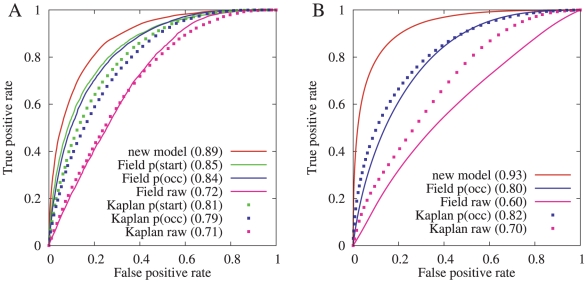
Classification performance comparisons. (A) Comparison in *S. cerevisiae* between our model and the models of Field *et al.* [4] and Kaplan *et al.* [10]. These two previously published models each produce three types of scores at each nucleotide: a raw binding score, a probability that a nucleosome starts at that position, and a nucleosome-occupancy probability. The *S. cerevisiae* dataset used in this evaluation contains the top-scoring 6,355 positions or approximately 1/8 of the entire dataset. (Top-scoring means most well-positioned based on experimental data, not highest pattern-correlation scores.) (B) Similar comparison in *H. sapiens* between our model and the models of Field *et al.* and Kaplan *et al.* The raw binding scores and the occupancy probabilities were downloaded from the Segal lab website. The *H. sapiens* dataset used in this evaluation contains 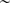
200,000 dyad positions.

We compared the predictive performance of our model to the two recently published nucleosome prediction models described by Field *et al.* [4] and by Kaplan *et al.* [10]. These two previously published models are algorithmically very similar, the main distinction being that the Field model was trained on *in vivo S. cerevisiae* mono-nucleosomes, while the Kaplan model was trained using a genome-wide occupancy map of nucleosomes assembled *in vitro* on purified *S. cerevisiae* genomic DNA. For comparisons in *H. sapiens*, we downloaded the occupancy probabilities and raw binding scores from the Segal lab website, and for comparisons in *S. cerevisiae*, we downloaded the executable and obtained raw binding scores, start probabilities and occupancy probabilities for the entire *S. cerevisiae* genome. Our model is a purely local scoring function, requiring only 301 bp of sequence to make a prediction at a single point, and as such, is computationally most similar to the raw binding scores from these two models. However, the Field and Kaplan raw binding scores are sensitive to variations in the local AT-content and are not able to discriminate accurately between, for example, nucleosome dyad positions in high-AT regions and linker positions in low-AT regions. The dynamic programming stage of the Field and Kaplan models corrects for this sensitivity, and the resulting occupancy and start probabilities are better able to discriminate between dyads and linkers. The ROC curves for the Field and Kaplan models on representative datasets from *S. cerevisiae* and *H. sapiens* are shown in Figure 7, together with the corresponding ROC curves for our model.

In all binary classification tests presented here, the positive examples are the experimentally determined dyad positions, while the negative examples are the positions 110 bp to either side of each dyad. This definition of negatives examples (*i.e.* linkers) is different from that used in [4] in which linkers were defined as contiguous regions of length 50–500 bp not covered by any nucleosome. We chose to use a different definition for two reasons: first, long nucleosome-free regions may be a result of the experimental protocol [31], [32], and second, sequencing-depth limitations in *H. sapiens* genome-wide experiments mean that true negatives are far outweighed by false negatives. Furthermore, the extent to which an individual dyad position can be distinguished from its immediately adjacent linker regions is a direct indication of the apparent positioning stringency. The difference in discrimination performance between our model and the two previous models is more significant in the *H. sapiens* dataset. This is to be expected as the Field and Kaplan models were both trained on *S. cerevisiae* datasets. In the *S. cerevisiae* evaluation (Figure 7a), the Field model outperforms the Kaplan model, which is also to be expected as it was trained on this test data (although the classification task here is different than that shown in [4] because we have defined the negative class differently). In Figure 7a, at a false positive rate of 10%, our model has a true positive rate in *S. cerevisiae* of 64% compared to true positive rates of 55%, 51% and 22% for the three Field scores, and 46%, 38%, and 22% for the three Kaplan scores. For the *H. sapiens* evaluation shown in Figure 7b, the Kaplan model outperforms the Field model. At a false positive rate of 10%, our model has a true positive rate of 79% compared to true positive rates of 49% and 26% for the two Kaplan scores, and 41% and 17% for the two Field scores.

**Figure 8:**
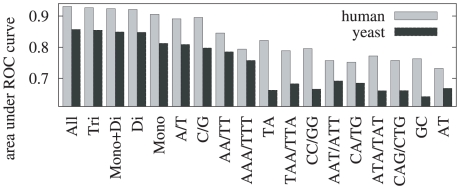
Classification performance of individual k-mers and subsets of k-mers. Area under the ROC curve obtained using features associated with individual k-mers as well as certain subsets of k-mers. *All* represents the set of all k-mers of length 1, 2, 3. *Tri* represents the set of all trinucleotides, *Di* the set of all dinucleotides, and *Mono* the set of mono-nucleotides. The features are ordered in the graph according to the average performance on *H. sapiens* and *S. cerevisiae*. All subsets perform better than any individual k-mer, and the most discriminative individual k-mers are the mono-nucleotides A/T and G/C, followed by the dinucleotide AA/TT and the trinucleotide AAA/TTT. This analysis is based on the top-scoring 12,698 *S. cerevisiae* positions, and the top-scoring 209,101 *H. sapiens* positions.

We further analyzed the performance of our model by considering different subsets of the features as well as features associated with individual k-mers in order to determine which features are most informative, and to investigate whether this was consistent between *H. sapiens* and *S. cerevisiae* (Figure 8). Not surprisingly, in general the more features are used, the better the performance, but the best individual features are the mono-nucleotides—and this is true for both *H. sapiens* and *S. cerevisiae*. This result is rather surprising and indicates that these mono-nucleotide patterns are able to summarize the relevant information in longer homo-polymer stretches. The most informative di- and tri-nucleotides are AA/TT and AAA/TTT, reconfirming the importance of poly(dA∶dT) tracts in the organization of nucleosomes [33].

### Analysis of model-predicted nucleosome repeat lengths

Nucleosomes are the basic repeat element of the first level of the chromatin structure, forming the “beads-on-a-string” fiber which in turn coils into a larger structure known as the 30 nm fiber. The average length of the linker DNA between adjacent nucleosomes defines the nucleosome repeat length which in turn affects the structure and size of the 30nm fiber [34], [35]. Because our model is essentially a pattern-matching algorithm, we were interested in evaluating whether the nucleosome pattern described by our model appeared to repeat at regular intervals along the *H. sapiens* and *S. cerevisiae* genomes.

**Figure 9:**
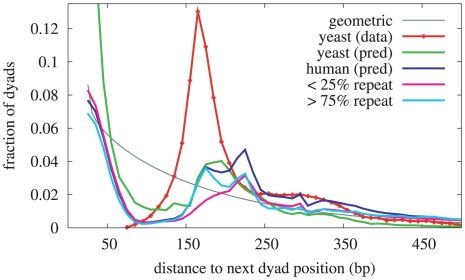
Distribution of distances between successive nucleosome dyad positions. The distributions shown here were derived from Field *et al.* [4] *S. cerevisiae* data (red), and from genome-wide model predictions in *S. cerevisiae* (green), and in *H. sapiens* (dark blue). The predicted dyad positions in *H. sapiens* are also shown partitioned according to the fraction of the neighboring 200 bases that are marked as repetitive (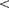
25% repeat in pink, and 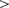
75% repeat in aqua). For the purposes of this analysis, a predicted dyad position is a local maximum in the dyad score trace. The grey line shows the geometric distribution resulting from random positions with an average spacing of 165 bp.

It is possible to obtain an empirical distribution of distances between successive experimentally-determined *S. cerevisiae* dyad positions because this set constitutes a significant fraction of the total number of nucleosomes expected in *S. cerevisiae*. The resulting distribution is shown in Figure 9 and confirms the dominant nucleosome repeat length of 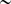
165 bp in *S. cerevisiae* [36]. The empirical distribution obtained from the far sparser set of experimentally-determined *H. sapiens* dyad positions is less reliable (in the largest set of over 800,000 positions, over 80% of the positions are more than 500 bp away from the nearest upstream position), but it too shows a clear peak—in this case at 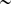
200 bp, although less than 2% of the dyad positions are between 190 and 210 bp from the nearest upstream position. We made genome-wide predictions for both *H. sapiens* and *S. cerevisiae* using our model, and found that the distribution of distances between successive predicted dyad positions (local maxima with positive model scores), showed interesting trends. For *S. cerevisiae*, aside from an over-representation of dyads predicted to be close together, there is a single broad peak between 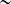
175 and 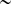
200 bp, while for *H. sapiens* there is a bimodal distribution with one peak at 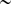
175, and one at 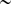
225 bp, as shown in Figure 9. If the local maxima were randomly distributed, the distribution of distances from one to the next would follow a geometric probability distribution, monotonically decreasing for longer inter-dyad distances.

**Figure 10:**
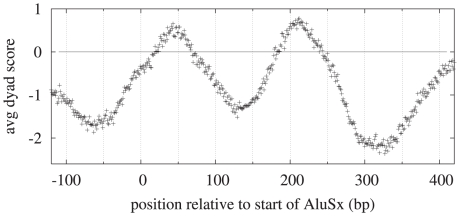
Average dyad scores for *AluSx* repetitive element. Dyad scores were computed for 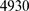 AluSx elements, including adjacent sequence, and then aligned at the start position and averaged. The model predicts locally optimal dyad positions at 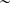
40 and 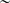
210 bp relative to the start of the 313 bp long repetitive element.

We hypothesized that repetitive sequences may be responsible for a significant number of consistent inter-dyad distances in *H. sapiens*, and found that partitioning the predicted dyad positions according to the local repeat content showed that the predicted dyads in repetitive regions contribute to both local maxima in the distance distribution, while the predicted dyads in non-repetitive regions contribute mainly to the second peak in the distance distribution. The relationship between *Alu* repeats and nucleosome formation has been widely studied—specifically, *Alu* sequences have been shown to facilitate the formation of nucleosomes *in vivo* [37], [38], with one putative nucleosome centered over the RNA polymerase III promoter A box (near the 5′ end of the *Alu* sequence), and a second putative nucleosome positioned over the right arm of the *Alu* element, flanked by two A-rich regions. Because the upstream and downstream sequence also affect our model predictions, we extracted DNA sequence surrounding 4930 separate *AluSx* sequences (the most common type of *Alu* repeat), and found that our model predicts two strong dyad positions 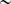
170 nucleotides apart, as shown in Figure 10. Our model's prediction of just two locally optimal dyad positions, which could be simultaneously occupied by a pair of adjacent nucleosomes, is in contrast with recently published predictions of several alternative nucleosome positions separated by multiples of 10.4 bases [39]. A nucleosome centered at position 40 would wrap the first 110 bp of the *Alu* sequence as well as approximately 30 upstream base pairs around the histone octamer, effectively blocking access to the two internal Pol III promoters and possibly an upstream enhancer, and rendering the *Alu* transcriptionally silent. We also analyzed all *H. sapiens* repetitive sequences in RepBase[40] (considering only the consensus sequence and disregarding flanking regions from specific instances of the repeat in the *H. sapiens* genome) and found that several longer repetitive elements resulted in predicted dyad positions at spacings between 165 and 185 bp, including endogenous retroviruses (ERV1, ERV2 and ERV3), non-LTR retrotransposons (L1 in particular), and DNA transposons (such as the *Mariner* transposable element).

### Predicted nucleosome positioning near strong boundary elements

Transcription start sites (TSS) and CTCF-binding sites have been shown to be strongly correlated with ordered arrays of nucleosomes based on numerous experimental assays. Using high-resolution tiling microarrays to analyze nucleosomal DNA in *S. cerevisiae*, Lee *et al.* [18] identified a general pattern of nucleosome occupancy anchored at the TSS. The average nucleosome occupancy signal, aligned at the TSS and averaged over all genes, shows a nucleosome free region centered approximately 30 bp upstream of the TSS, flanked by the −1 nucleosome centered 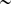
170 bp upstream of the TSS and the +1 nucleosome centered 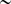
100 bp downstream. The strict positioning of nucleosomes further upstream and downstream from the TSS decays gradually, although more slowly in the transcribed region (downstream). A statistical packing model of nucleosome positioning was proposed by Mavrich *et al.* [14] whereby the genomic sequence specifies the locations of the +1 and −1 nucleosomes, and these strictly positioned nucleosomes maintain a relatively large nucleosome-free region over the TSS, acting as barriers against which adjacent nucleosomes are packed. Using the Barski ChIP-seq dataset, Zhang *et al.* showed similar results in *H. sapiens*, with the +1 nucleosome again the most strongly positioned, just downstream of the TSS [2]. Using the same approach, they observed 3-4 well positioned nucleosomes on either side of CTCF binding sites, while the binding site itself showed strong depletion. Further studies of nucleosome organization surrounding CTCF binding sites have suggested that binding of CTCF provides an anchor point for positioning nucleosomes [41], [42], while unbound sites are occluded and rendered inaccessible by the presence of a nucleosome [42]. We note, however, that recent studies have shown that regions that have been previously described as ‘nucleosome-free’ are in fact frequently occupied by nucleosomes that are unusually unstable under the conditions normally used in sample preparation [31], [32].

In order to evaluate to what extent our model replicates these experimental results for transcription start sites, we applied our scoring method to a set of *S. cerevisiae* DNA segments aligned at 5,015 high-confidence TSS and clustered according to promoter nucleosome signatures as in [18] (Figure S10), and to a set of *H. sapiens* DNA segments aligned at the 32,079 TSS from the DBTSS database of *H. sapiens* transcriptional start sites [43] (Figure S11a). The *S. cerevisiae* TSS predictions agree with the experimental results in the cluster-dependent strength of the nucleosome-free region upstream of the TSS, and indicate that the sequences associated with clusters identified experimentally also result in different average model predictions. The *H. sapiens* TSS predictions are dominated by the +1 nucleosome dyad peak 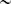
65 bp downstream of the TSS, with two additional peaks clearly visible further downstream. Unlike the *S. cerevisiae* TSS predictions, which show evidence of a larger than average linker between the +1 nucleosome at 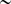
60 bp downstream of the TSS and the −1 nucleosome at 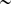
190 bp upstream, the *H. sapiens* TSS predictions suggest the presence of a weakly positioned “0” nucleosome 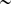
105 bp upstream of the TSS, between the −1 nucleosome (at approximately −270 bp) and the +1 nucleosome (at approximately +65 bp). We caution, however, that computing average profiles by averaging these locally-computed scores over a set of aligned DNA sequences has inherent drawbacks in that the resulting average will be dominated by the sequences with the highest-scoring dyad locations (and the lowest-scoring linker locations) as well as by the relative positions of these peaks (and troughs). Posterior probabilities of nucleosome occupancy, obtained by post-processing these local scores using a dynamic programming approach may result in average profiles that more closely reproduce experimental results. Indeed, such average profiles, representing thousands of TSS, whether based on predictions or on experimental data, fail to convey the substantial variation that exists in the position of the +1 nucleosome. In order to confirm that our model accurately reproduces this variation, we formed subsets of the DBTSS sites according to the position of the “+1” nucleosome in the Zhang dataset (if any), using 30 bp windows spanning the region from 40 bp upstream of the TSS to 200 bp downstream, and computed average predictions for each of these subsets. We found that the average predicted position of the +1 nucleosome for each of these subsets correlates extremely well (R = 0.99) with the average experimentally-inferred position. (See Figure S11b for three representative subsets.)

We similarly aligned and analyzed a pair of CTCF-binding site datasets [41]: a set of 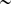
6000 occupied binding sites and a set of 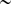
6000 unoccupied sites (Figure S12). The average model scores near CTCF binding sites indicate that the binding site itself is a favorable nucleosome dyad position, in agreement with the experimental observation that unbound sites are occluded by nucleosomes [42]. These predictions are similar to those in [15], although our method produces a significantly narrower peak at the binding site. On either side of the CTCF binding site, at 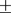
300 bp are two weak peaks in the average dyad score, but the regular pattern of nucleosomes on either side of occupied CTCF sites that has been observed experimentally [41], [42] is not matched by the predictions, suggesting that the positioning of nucleosomes near occupied CTCF sites is driven primarily by statistical packing against a barrier.

## Discussion

The nucleosome DNA pattern that we have described here is largely consistent with previously described nucleosome positioning signals. However, our finding that the two mono-nucleotide patterns (A/T and G/C) are individually more predictive than any single dinucleotide or trinucleotide pattern represents a significant departure from the widely held belief that dinucleotide periodicities and poly-A/T tracts are the strongest nucleosome positioning elements. Our model agrees with the hypothesis that periodicity seen in the average profile of a set of nucleosomal sequences reflects an alignment imposed by the structural organization of the nucleosome core particle, rather than periodicity in individual sequences [27]. Elevated GC-content is widely known to be a key feature of nucleosomal sequences and was previously found to be one of the strongest individual predictors of nucleosome occupancy in *S. cerevisiae* [16], [18]. GC-rich dinucleotides have also been associated with reduced DNA deformation energy which would facilitate their integration into the core of the nucleosome [44], [45]. The downward trend 5′ to 3′ across the nucleosome core of the AA dinucleotide frequency has been previously observed [14], [26], but the significance of this asymmetry in localizing nucleosomes has not been emphasized.

The symmetry of the nucleosome around the dyad axis and the reverse-complementarity of the two strands of the double helix require that the A and T patterns form a mirror-image pair, and likewise for G and C. If each individual pattern was symmetric around the dyad axis, then only two distinct patterns would exist: one for A/T and one for G/C. Furthermore, because p(A+T) and p(G+C) must sum to unity, these two patterns would be perfectly negatively correlated, and from an information-theoretic point of view the second pattern would provide no additional information not already available in the first. Because each individual pattern is *not* symmetric around the dyad axis, the four mono-nucleotide patterns combine to provide more information regarding the locally optimal dyad position. We verified this by mapping the DNA sequences down to a two-letter 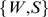 alphabet and training the model as before, and found that the discrimination performance as measured by the area under the ROC curve was significantly reduced.

Our model combines the features that promote nucleosome occupancy as well as those that enforce exclusion into a set of k-mer specific patterns. The pattern-correlation method that we use is normalized to remove sequence composition biases, as is also done in the nucleosome-core portions of the Field and Kaplan models [4], [10]. However, the widening of the model to include the adjacent linkers in each pattern similarly removes composition bias from the scoring of the linkers, resulting in an overall description of the nucleosome that is insensitive to large-scale variations in AT content, an insensitivity which naturally reflects the pervasive presence of nucleosomes in all genomic regions. Our approach also combines elements of previous probabilistic models [4], [9], [10], [13], with a discriminative approach [16], [17]. This weighted combination of features allows us to simultaneously make use of mono, di- and tri-nucleotide patterns which provide complementary information. Ioshikhes *et al.* [13] modeled only the distribution of AA and TT dinucleotides, effectively giving zero weight to all other dinucleotides. Our approach is a generalization of this idea, and we confirm that, of the 10 unique dinucleotides, AA/TT is the most predictive of nucleosome position, while AC/GT and GA/TC are the least predictive.

Examining distances between predicted dyad positions on a genome-wide scale, we find evidence for two classes of preferred nucleosome repeat lengths in *H. sapiens*—one near 175 bp and the other near 225 bp. In *S. cerevisiae*, a similar analysis produces a broad peak between 175 and 200 bp. This predicted distribution in *S. cerevisiae* implies longer linkers than the experimentally inferred distribution, a bias toward longer nucleosome repeat lengths which may be caused by the length of our pattern. Although the pattern length of 301 bp was chosen to optimize performance on our datasets of nucleosome positions, the original experiments themselves and the post-processing of the data to obtain estimated dyad positions may produce an ascertainment bias that favors not only highly-positioned nucleosomes but also those flanked by longer linkers. In the *H. sapiens* genome, the shorter class of linkers are associated with repetitive elements while the longer class of linkers are associated with both repetitive and non-repetitive elements. We hypothesize that, by preferring two different classes of linker lengths, the repetitive elements promote the formation of the two distinct classes of 30 nm chromatin fiber described by Robinson *et al.* [24].

The processing of the high-throughput sequencing data ensures that only the most stringently positioned nucleosomes will result in high-confidence dyad positions. The largest *S. cerevisiae* dataset we considered contained approximately 50,000 nucleosome positions, or nearly 70% of the expected total number of nucleosomes within the *S. cerevisiae* genome. By contrast, the largest *H. sapiens* dataset we considered contained only 5% of the 15,000,000 nucleosomes we estimate would be required by a single copy of the *H. sapiens* genome. Our model achieved similar performance on two pairs of datasets: the 3,000 *S. cerevisiae* nucleosomes and 400,000 *H. sapiens* nucleosomes, and the 6,000 *S. cerevisiae* nucleosomes and 800,000 *H. sapiens* nucleosomes. For the smaller pair of datasets, we report a true positive rate of 74% at a false positive rate of 10%, and for the larger datasets, we report a true positive rate of 65% at a false positive rate of 10%. If we assume that roughly half of the well-positioned nucleosomes in *H. sapiens* were missed through a combination of issues due to short-read sequence mappability and sequencing-depth limitations, then these two pairs of datasets represent 4% and 8% respectively of the entire set of nucleosome positions for these two genomes. This implies that, in both genomes, a comparable and relatively small fraction of nucleosomes are well-positioned and that these positions are predictable based on sequence alone. We believe that the bulk of the remaining nucleosomes follow a statistical positioning model [14]. Our results lead us to a middle ground between, on the one hand, the idea that nucleosome positions *in vivo* are determined primarily by DNA sequence [9], [10], and, on the other, the idea that intrinsic histone-DNA interactions play no part in creating the *in vivo* pattern [46]. Nucleosome occupancy models in which short linkers are preferred [19] may predict certain nucleosomes to be well-positioned not as a result of a strong local sequence signal, but rather as a direct result of a nearby nucleosome that is itself positioned by a particularly strong sequence signal, the effects of which ripple outwards in the chromatin structure. Knowing which nucleosomes are strongly positioned due to local sequence signals and which ones are merely “packed” against a barrier would further our understanding of the organization of the chromatin.

Our estimates that relatively small fractions of nucleosomes are strongly-positioned based on local sequence alone may seem surprising in light of some earlier claims that 50% or more of nucleosome positions could be accurately predicted based on sequence alone [9], [26]. However, these earlier claims were based on very small sets of well-positioned nucleosomes (a few hundred as opposed to tens or hundreds of thousands), or on criteria which could be satisfied for 32–45% of nucleosome positions by chance. We have defined a more stringent classification task and have tried to assess the fraction of nucleosome positions that are strongly influenced by local sequence features.

Further avenues for research to improve this model include discriminatively combining patterns of different lengths or different horizontal scales to capture the variation in linker lengths, as well as investigating the possibility that different types of nucleosomes may be associated with different DNA sequence patterns—for example, a difference in the GC profile of H2A.Z nucleosomes has been recently described [27]. Another interesting direction is to use these dyad scores as well as the experimentally estimated nucleosome positions to train a dynamic Bayesian network which could then be used to make nucleosome-positioning and occupancy predictions. In addition, these predictions could be constrained by the experimental evidence and used to fill in gaps in the data.

## Methods

### Dyad position estimation from sequencing

The dyad positions in *H. sapiens* were estimated using a modified version of the NPS (Nucleosome Position from Sequencing) software [2]. The original implementation combines offset tags from each strand into a smoothed nucleosome occupancy trace. A p-value threshold is applied to this trace, and the end-points of the regions that exceed the threshold (with boundaries on the minimum and maximum region extents) are called positioned nucleosome regions. Our initial pattern was obtained using the Zhang nucleosome positions by assuming that the dyad was at the mid-point of each of these nucleosome regions. The modified NPS software finds the local maximum within each region that crosses the threshold and calls that the dyad position.

To estimate dyad positions from the Field dataset of mapped reads [4], each mapped read was represented on the genomic axis by a triangle of height 1, and base given by the length of the actual read, and these overlapping triangles were summed to produce a “dyad” trace. All local maxima within local windows of length 141 nucleotides were called dyad positions.

### Nucleosome sequence pattern estimation

Given a set of 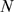 nucleosome dyad positions and a k-mer 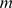 of length 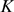, we compute the *m*-pattern 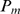 in four steps as follows. First, extract a DNA segment 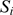 of width 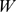 (where 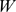 is odd) centered at each dyad position 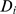 from the reference genome. Second, for 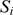 and its reverse complement 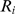, convert the DNA segment into a numerical representation in which all positions are zeros except each position of an exact sub-string match to 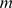 is set to the value 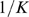. If 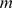 is a mono-nucleotide, then 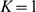, and the numerical representation is a simple bit vector of 1's and 0's. For longer 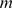 it is possible to have overlapping matches (*e.g.* the dinucleotide AA occurs four times with overlap in the segment 5-mer AAAAA), and in such cases the values are summed. The sum of the 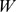 values in the resulting numerical representation is equal to the number of (possibly overlapping) occurrences of 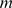 in the input DNA segment and its reverse complement. Third, average all 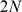 numerical representations to obtain the average pattern, and finally standardize this pattern such that its mean is equal to zero and its variance is equal to 1. This procedure (excluding the standardization step) is expressed in the following equation:
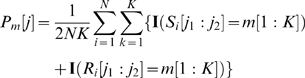
where 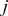 is the position relative to the dyad and ranges from 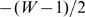 to 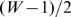, 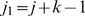, 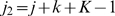, 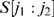 indicates the substring in 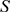 from position 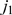 through position 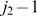 inclusive, and 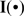 is the indicator function which is equal to 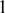 if the argument is TRUE, and 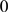 otherwise. This procedure will produce mirror-image patterns for k-mers that are reverse-complements of one another. For example, the pattern 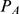 for the mono-nucleotide A, and pattern 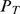 for the mono-nucleotide T, will be related as follows: 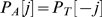, for 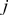 as defined above. Dinucleotides, such as TA or GC, which are their own reverse-complements will result in symmetric patterns for which 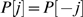.

### 3D structure visualization

The 3D visualizations of the nucleosome core particle shown in Figure 2 were created using PyMol [47] and PolyView-3D [48] and PDB [49] structure 1KX5 [50].

### Null model estimation

Our null model for the variation expected by chance of each nucleotide across a distance of 151 bp was derived empirically from 800,000 random sets of DNA sequence fragments. Each random set is equal in size (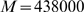) to the Zhang positions set: for each nucleosome dyad position 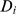, we choose a random position 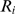 within 1000 bp (in either the 3′ or the 5′ direction). We then extract a set of 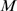 DNA sequences of length 151 bp centered at each of the random positions 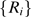, and construct the position specific frequency matrix (PSFM) as described earlier. We search for the absolute maximum and minimum values for each nucleotide across the 151 bp PSFM, and compute the difference, 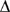. For each of the four traces in the observed pattern shown in Figure 1 (top), 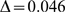. Our empirical null model is shown in Figure S3. The right tail of the empirical null model falls off proportional to 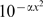 based on which we estimate the probability of observing a 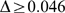 by chance to be 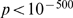.

### DNA sequence pattern correlation

Given the pre-computed pattern 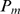 for the k-mer 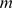 and a new input DNA sequence 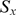 of length 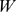, we start by translating both 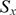 and its reverse-complement 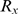 into the numerical representations 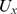 and 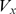 according to *m*:
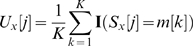


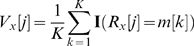
Both 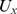 and 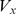 are then standardized to have mean zero and variance one, and the sum of the dot-products between each of these vectors and the pre-computed pattern vector is our correlation coefficient:
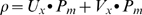
This correlation coefficient represents how well the input DNA sequence pattern 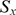 matches the patterns for both the k-mer 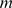 and its reverse complement.

### Support vector machine training and testing

Given a set of positive and negative examples described by feature vectors 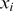 of length 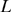, a support vector machine (SVM) learns an optimal *discriminant function* defined by a weight vector 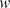 such that the dot-product 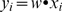 will “best” separate the positive examples (with 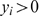) from the negative examples (with 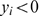). If the positive examples cannot be separated from the negative examples by a hyperplane in the high-dimensional feature space, 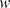 will define the hyperplane that minimizes the misclassification costs [30].

### Evaluation using ROC curves, and evaluation of individual features or subsets of features

ROC curves provide a means of evaluating a binary classifier. A set of positive examples and a set of negative examples are assigned scores, and the examples are then ordered from highest score down to lowest. The ROC curve is generated by varying a threshold from the minimum score up to the maximum score, and at each step computing the true positive rate (the fraction of positives scoring above the threshold) and the false positive rate (the fraction of negatives scoring below the threshold). A perfect classifier will result in a line from (0,0) up to (0,1) and across to (1,1), and an area “under” the curve (AUC) of 1.0, while a random classifier will result in a diagonal line from (0,0) to (1,1), and an AUC of 0.5.

For our classification tasks, the negative examples were defined to be at a distance of 110 bp on either side of each positive example, resulting in twice as many negative examples as positive examples.

When evaluating the Field and Kaplan models, start probabilities were converted to dyad probabilities by shifting the predictions by 73 bp. The raw binding scores were locally averaged over a window of width 9, and the start probabilities were locally averaged over a window of width 41—these window sizes were chosen to optimize the area under the ROC curve. Our model predictions were locally averaged over a window of width 11.

### Partitioning according to local repeat content

For the “
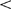
25% repeat” and “
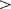
75% repeat” curves in Figure 9, dyads were partitioned into sets according to the fraction of bases annotated as repetitive by RepeatMasker [51] within a 200 bp window centered at the dyad. For the “
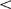
25% repeat” curve, only those dyads with fewer than 50 out of 200 bp marked as repetitive were considered to compute the inter-dyad distance histogram. Similarly, for the “
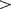
75% repeat” curve, only those dyads with more than 150 out of 200 bp marked as repetitive were considered.

### AluSx analysis

There are over 340,000 AluSx elements annotated by RepeatMasker [51], with lengths generally between 291 and 313. In order to perfectly align a large set of AluSx sequences with flanking regions, we used only the 4,930 AluSx sequences of length 313 with no insertions or deletions. We then extracted 901 bp of sequence surrounding the mid-point of each of these AluSx sequences and computed the dyad score along each of these sequences. The average of these dyad score traces is shown in Figure 10.

## Supporting Information

Table S1 Breakdown of nucleosome position sets and ROC scores by chromosome for *H. sapiens*. The *All* dataset was obtained using a threshold of 1.e-05 on the NPS-assigned p-value, and contains the top 828,883 scoring nucleosome dyad positions obtained from the Schones resting T-cells dataset. The *Top 1/2* dataset was obtained by lowering the threshold to 1.e-08, and contains 398,291 dyad positions, and the *Top 1/4* dataset was obtained by further lowering the threshold to 1.e-11, and contains 209,101 dyad positions. For each chromosome and for each set, the number of positions in that set and on that chromosome is given, followed by the *density* of positions estimated simply as the chromosome length divided by the total number of positions, followed by the cross-validated area under the ROC curve obtained by training on all other chromosomes. Note that both X and Y are significantly under-represented in terms of nucleosome positions as compared to the autosomes. The performance as measured by the area under the ROC curve is very consistent across all of the chromosomes except Y.
(0.01 MB PDF)

Table S2 Breakdown of nucleosome position sets and ROC scores by chromosome for *S. cerevisiae*. This table is very similar to the one on the previous page for *H. sapiens*. The *All* dataset (far right) represents all of the nucleosome dyad positions inferred from the Field *et al.* data [4]. Moving from right to left, each successive dataset contains the top-scoring half of the dataset to the right.
(0.01 MB PDF)

Figure S1 Histogram of distances between short-read tags mapped to the top- and bottom-strands for the composite Barski data set and the Schones data set. The histogram consists of counts in bins of width 10 nucleotides, and the y-axis is normalized by the count in the first bin. The NPS algorithm looks for regions in which sets of plus-strand tags and minus-strand tags are separated by a distance that corresponds to a single nucleosome core. Based on the analysis shown in this figure, it is clear that the Schones data set provides a more consistent set of tags separated by a distance of approximately 140 nucleotides. The agglomeration of the 21 separate ChIP-Seq experiments in the Barski data set is less enriched for tags separated by the expected distance, and the observed spread is significantly wider. Based on this analysis, we chose to proceed with our analysis using only the Schones data set.
(0.01 MB PDF)

Figure S2 Single-nucleotide PSFM computed across all nucleosome sequences, using only the top-strand sequence centered at each nucleosome dyad. The bottom figure zooms in on the 300 positions centered at the dyad. The gradual increase in the total GC-content shown in Figure∼S1 as the distance to the nucleosome dyad decreases is due to a relative over-representation of nucleosome positions in the Zhang dataset in GC-rich regions of the genome. This over-representation of nucleosome positions in GC-rich regions is also described by Zhang *et al.* [2] and is attributed to a combination of the ChIP-selection for histone modifications that are known to be over-represented in genes and near promoters, and the known GC-bias in the coverage of Solexa sequencing.
(0.10 MB PDF)

Figure S3 The x-axis represents the maximum absolute variation observed in a mono-nucleotide pattern derived by averaging 438,000 individual DNA sequences of length 151 bp each. The null-model distribution was obtained empirically from 800,000 random sets of DNA fragments. (a) shows the null model distributions based on random sampling for each of the 4 nucleotides as well as the observed deltas based on the nucleosome pattern derived from the Zhang positions, and (b) shows the null distributions at higher resolution.
(0.01 MB PDF)

Figure S4 The nucleosome positions are over-represented in GC-rich regions, but the pattern is observed across different ranges of AT content and is strongest in the very high AT content subset (c) and weakest in the very low AT content subset (f). (a) shows the pattern derived from all of the nucleosome positions and (b) through (f) show the patterns derived from non-overlapping quintiles, divided according to AT content. (For each plot, A (green) and T (red) are plotted against the y-axis on the left, while C (blue) and G (yellow) are plotted against the y-axis on the right.)
(0.04 MB PDF)

Figure S5 The nucleosome pattern is largely unchanged when the dataset is partitioned according to local repeat content, although the A and T curves shift upwards relative to the C and G curves. (a) 70% of the nucleosome positions are at least 30 bases away from a repeat; (b) 19% are in a repeat that extends at least 30 bases to both sides; and (c) 11% are near the edge of a repeat. The Pearson correlation between local AT content and local repeat fraction is +0.23 (although Alu repeats are only 45–50% AT, other repeats generally have higher AT content, particularly LINE1 repeats which are approximately 65% AT). The increased noise in the patterns in (b) and (c) is due to the smaller number of sequences used to create these two patterns.
(0.02 MB PDF)

Figure S6 The four possible permutations of the original pattern were correlated against all nucleosome sequences. Each sequence was then assigned to one of four classes, and new patterns derived after realigning the sequences according to the peak correlation offset (green = A, red = T, blue = C, yellow = G).
(0.03 MB PDF)

Figure S7 A subset of the nucleosome patterns derived from the training set of ∼200,000 nucleosome dyad positions. The pattern for T is a reflection of the A pattern across the (vertical) dyad axis, and similarly the TT pattern is a mirror image of the AA pattern. (The same holds for C/G and CC/GG.) The patterns for dinucleotides which are their own reverse complements are symmetrical about the dyad axis. Intriguingly, the normalized patterns for CG and GC are nearly identical to one another, suggesting that these dinucleotides play the same role in positioning nucleosomes despite their dramatically different occurrence rates.
(0.02 MB PDF)

Figure S8 These figures show the *S. cerevisiae* and *H. sapiens* nucleosome patterns for A, AA, and AAA. Note that these *S. cerevisiae* patterns still contain the MNase artifact, which was most apparent in the A pattern. The Pearson correlation coefficients for each *S. cerevisiae* pattern and the corresponding *H. sapiens* pattern are: A:0.77, AA:0.88, and AAA:0.91, suggesting that the underlying oscillation common to both patterns plays a role in positioning nucleosomes across species.
(0.02 MB PDF)

Figure S9 Area under the ROC curve as a function of the distance *L* between the dyad positions and the *non*-dyad positions on either side. Near the dyad, discrimination is difficult and the performance is not much better than random, especially in *H. sapiens*. As the distance from the dyad increases, performance improves, reaching a maximum in the linker, after which the performance degrades again.
(0.01 MB PDF)

Figure S10 These four traces represent the average dyad score across four subsets of *S. cerevisiae* transcription start sites, clustered according to promoter nucleosome profile by Lee *et al.* [18]. The grey curves accompanying each of the dyad score traces were taken from Figure 4a in the same paper by Lee *et al.* (Note that the grey traces are not plotted on the same y-axis scale as the average dyad score traces, but are plotted on the same scale relative to each other.) The GO Slim biological process term most overrepresented by genes in each cluster, as reported by Lee *et al.* were: “response to stress” (top, red); “translation” (next, green); “ribosome biogenesis and assembly” (next, dark blue); and “organelle organization and biogenesis” (bottom, light blue).
(0.02 MB PDF)

Figure S11 (A) Model predictions averaged over 32,000+ transcription start sites from DBTSS (red), and experimentally derived dyad curve based on the Zhang set of nucleosome positions (grey). Note the ∼40bp offset between the red and grey curves, and the prediction of a nucleosome near −100 bp, in the apparent “nucleosome free region’ (NFR) upstream of the TSS. The apparent disagreement between the predictions and the experimental averages are due to two effects: first, the experimental data only spans approximately one third of the entire set of 32,000 TSSs; and second, the locally optimal dyad score for each +1 nucleosome (for example) may vary considerably, thereby contributing unequally to the average profile. In order to verify that the ∼40 bp offset did not represent a systematic bias in our method, we created subsets of the DBTSS positions according to the relative position of an experimentally-determined dyad. We then computed the mean predicted dyad score across these subsets. Three representative subsets are shown in (B): the number of TSS in each subset is indicated by the number in the parentheses, and the mid-point of the 30 bp window is given by the number preceding the parentheses. For example, the red curve represents an average over 1252 predictions, for transcription start sites with experimental dyad positions mapped between 20 and 50 bp downstream of the TSS. The position of the peak in each of these average curves matches the mid-point of the 30 bp window almost exactly. (The grey curve in (A) is not plotted on the same y-axis as the red curve and is shown only for reference.)
(0.02 MB PDF)

Figure S12 Model predictions averaged over two sets of CTCF binding sites: occupied (red) and unoccupied (green). The solid grey trace represents the experimentally-determined nucleosome occupancy derived from Figure 5B in Cuddapah *et al.* [42] near unbound CTCF binding sites, and the thin grey line with triangles is the nucleosome occupancy curve surrounding bound CTCF sites from Figure 3A in Zhang *et al.* [2]. Without CTCF binding, the sequence-dependent nucleosome-positioning does not result in a strong coherent pattern of flanking nucleosomes, in agreement with the data from Cuddapah *et al.* In contrast, CTCF binding results in a significantly more coherent pattern of flanking nucleosomes. The difference between the red and the green curves suggests that the set of “unoccupied” CTCF sites includes a number of false-positives which appear to have significantly different flanking sequence characteristics. (The grey curves are not plotted on the same y-axis as the red and green curves and are shown only for reference.)
(0.02 MB PDF)
