# Supplementary material for: Learning a Weighted Sequence Model of the Nucleosome Core and Linker Yields More Accurate Predictions in Saccharomyces cerevisiae and Homo sapiens
Source: PLoS Comput Biol. 2010 Jul 8;6(7):e1000834. doi: 10.1371/journal.pcbi.1000834 (PMC2900294; doi:10.1371/journal.pcbi.1000834)
Supplement: Table S1 — Breakdown of nucleosome position sets and ROC scores by chromosome for H. sapiens. The All dataset was obtained using a threshold of 1.e-05 on the NPS-assigned p-value, and contains the top 828,883 scoring nucleosome dyad positions obtained from the Schones resting T-cells dataset. The Top 1/2 dataset was obtained by lowering the threshold to 1.e-08, and contains 398,291 dyad positions, and the Top 1/4 dataset was obtained by further lowering the threshold to 1.e-11, and contains 209,101 dyad positions. For each chromosome and for each set, the number of positions in that set and on that chromosome is given, followed by the density of positions estimated simply as the chromosome length divided by the total number of positions, followed by the cross-validated area under the ROC curve obtained by training on all other chromosomes. Note that both X and Y are significantly under-represented in terms of nucleosome positions as compared to the autosomes. The performance as measured by the area under the ROC curve is very consistent across all of the chromosomes except Y. (0.01 MB PDF) [file pcbi.1000834.s001.pdf]

chr	length (Mb)	Top 1/4			Top 1/2			All		
		count	density	ROC	count	density	ROC	count	density	ROC
1	247.25	19330	12791	0.93	36152	6839	0.91	73771	3352	0.89
2	242.95	19408	12518	0.93	36990	6568	0.92	75766	3207	0.89
3	199.50	15036	13268	0.93	28551	6988	0.91	59340	3362	0.88
4	191.27	11015	17365	0.93	21189	9027	0.91	45342	4218	0.89
5	180.86	12799	14131	0.93	24750	7307	0.91	51586	3506	0.89
6	170.90	12602	13561	0.93	23730	7202	0.92	48999	3488	0.89
7	158.82	10733	14797	0.93	20514	7742	0.92	43366	3662	0.89
8	146.27	10949	13360	0.93	20992	6968	0.92	43742	3344	0.90
9	140.27	8846	15857	0.93	17061	8222	0.91	35357	3967	0.89
10	135.37	11933	11345	0.93	22411	6041	0.92	45211	2994	0.89
11	134.45	11231	11972	0.93	21317	6307	0.91	44046	3053	0.89
12	132.35	9725	13609	0.93	18577	7124	0.92	38729	3417	0.89
13	114.14	6030	18929	0.94	11481	9942	0.92	24613	4638	0.90
14	106.37	6418	16573	0.93	12262	8675	0.91	25982	4094	0.89
15	100.34	6798	14760	0.93	12983	7728	0.91	26817	3742	0.89
16	88.83	6563	13535	0.93	12304	7219	0.92	25588	3471	0.89
17	78.77	6573	11985	0.94	12551	6276	0.92	25983	3032	0.90
18	76.12	5705	13342	0.94	10988	6927	0.93	23021	3306	0.90
19	63.81	3241	19689	0.93	6221	10257	0.92	13357	4777	0.90
20	62.44	6006	10396	0.94	11428	5463	0.92	23375	2671	0.90
21	46.94	2568	18280	0.92	4810	9760	0.91	10066	4664	0.90
22	49.69	3359	14794	0.94	6529	7611	0.92	13717	3623	0.90
X	154.91	1716	90276	0.93	3905	39671	0.93	10402	14893	0.91
Y	57.77	517	111747	0.64	595	97097	0.65	707	81716	0.67
ALL	3080.42	209101	14732	0.93	398291	7734	0.92	828883	3176	0.89

Table S1: Breakdown of nucleosome position sets and ROC scores by chromosome for *H. sapiens*. The *All* dataset was obtained using a threshold of 10^{-5} on the NPS-assigned p-value, and contains the top 828,883 scoring nucleosome dyad positions obtained from the Schones resting T-cells dataset. The *Top 1/2* dataset was obtained by lowering the threshold to 10^{-8}, and contains 398,291 dyad positions, and the *Top 1/4* dataset was obtained by further lowering the threshold to 10^{-11}, and contains 209,101 dyad positions. For each chromosome and for each set, the number of positions in that set and on that chromosome is given, followed by the *density* of positions estimated simply as the chromosome length divided by the total number of positions, followed by the cross-validated area under the ROC curve obtained by training on all other chromosomes. Note that both X and Y are significantly under-represented in terms of nucleosome positions as compared to the autosomes. The performance as measured by the area under the ROC curve is very consistent across all of the chromosomes except Y.
