# Supplementary material for: Learning a Weighted Sequence Model of the Nucleosome Core and Linker Yields More Accurate Predictions in Saccharomyces cerevisiae and Homo sapiens
Source: PLoS Comput Biol. 2010 Jul 8;6(7):e1000834. doi: 10.1371/journal.pcbi.1000834 (PMC2900294; doi:10.1371/journal.pcbi.1000834)
Supplement: Table S2 — Breakdown of nucleosome position sets and ROC scores by chromosome for S. cerevisiae. This table is very similar to the one on the previous page for H. sapiens. The All dataset (far right) represents all of the nucleosome dyad positions inferred from the Field et al. data [4]. Moving from right to left, each successive dataset contains the top-scoring half of the dataset to the right. (0.01 MB PDF) [file pcbi.1000834.s002.pdf]

chr	length (Kb)	Top 1/16			Top 1/8			Top 1/4			Top 1/2			All		
		count	density	ROC	count	density	ROC	count	density	ROC	count	density	ROC	count	density	ROC
1	230.2	88	2616	0.87	136	1693	0.87	237	971	0.83	433	532	0.76	843	273	0.72
2	813.2	217	3747	0.91	425	1913	0.88	872	933	0.85	1777	458	0.80	3512	232	0.73
3	316.6	109	2905	0.87	200	1583	0.88	359	882	0.85	666	475	0.82	1271	249	0.76
4	1531.9	359	4267	0.92	705	2173	0.88	1433	1069	0.86	3003	510	0.81	6331	242	0.73
5	576.9	143	4034	0.91	305	1891	0.87	627	920	0.84	1264	456	0.81	2449	236	0.74
6	270.2	71	3805	0.93	148	1825	0.91	303	892	0.86	591	457	0.79	1114	243	0.73
7	1090.9	249	4381	0.91	525	2078	0.89	1082	1008	0.86	2242	487	0.81	4658	234	0.73
8	562.6	177	3179	0.93	337	1670	0.88	647	870	0.84	1187	474	0.80	2308	244	0.73
9	439.9	133	3307	0.91	256	1718	0.88	520	846	0.85	994	443	0.80	1857	237	0.74
10	745.7	225	3314	0.91	450	1657	0.89	857	870	0.86	1676	445	0.81	3144	237	0.74
11	666.5	171	3897	0.94	357	1867	0.89	748	891	0.85	1507	442	0.81	2958	225	0.74
12	1078.2	274	3935	0.94	532	2027	0.91	1087	992	0.86	2197	491	0.81	4398	245	0.75
13	924.4	236	3917	0.91	482	1918	0.89	963	960	0.84	1938	477	0.79	4017	230	0.73
14	784.3	253	3100	0.91	493	1591	0.88	920	853	0.84	1723	455	0.80	3336	235	0.73
15	1091.3	267	4087	0.91	550	1984	0.89	1093	998	0.86	2271	481	0.81	4648	235	0.74
16	948.1	208	4558	0.92	454	2088	0.90	950	998	0.86	1915	495	0.81	3971	239	0.74
ALL	12070.9	3180	3796	0.91	6355	1899	0.89	12698	951	0.85	25384	476	0.81	50815	238	0.74

Table S2: Breakdown of nucleosome position sets and ROC scores by chromosome for *S. cerevisiae*. This table is very similar to the one on the previous page for *H. sapiens*. The *All* dataset (far right) represents all of the nucleosome dyad positions inferred from the Field *et al.* data [4]. Moving from right to left, each successive dataset contains the top-scoring half of the dataset to the right.
