# Supplementary material for: Learning a Weighted Sequence Model of the Nucleosome Core and Linker Yields More Accurate Predictions in Saccharomyces cerevisiae and Homo sapiens
Source: PLoS Comput Biol. 2010 Jul 8;6(7):e1000834. doi: 10.1371/journal.pcbi.1000834 (PMC2900294; doi:10.1371/journal.pcbi.1000834)
Supplement: Figure S1 — Histogram of distances between short-read tags mapped to the top- and bottom-strands for the composite Barski data set and the Schones data set. The histogram consists of counts in bins of width 10 nucleotides, and the y-axis is normalized by the count in the first bin. The NPS algorithm looks for regions in which sets of plus-strand tags and minus-strand tags are separated by a distance that corresponds to a single nucleosome core. Based on the analysis shown in this figure, it is clear that the Schones data set provides a more consistent set of tags separated by a distance of approximately 140 nucleotides. The agglomeration of the 21 separate ChIP-Seq experiments in the Barski data set is less enriched for tags separated by the expected distance, and the observed spread is significantly wider. Based on this analysis, we chose to proceed with our analysis using only the Schones data set. (0.01 MB PDF) [file pcbi.1000834.s003.pdf]

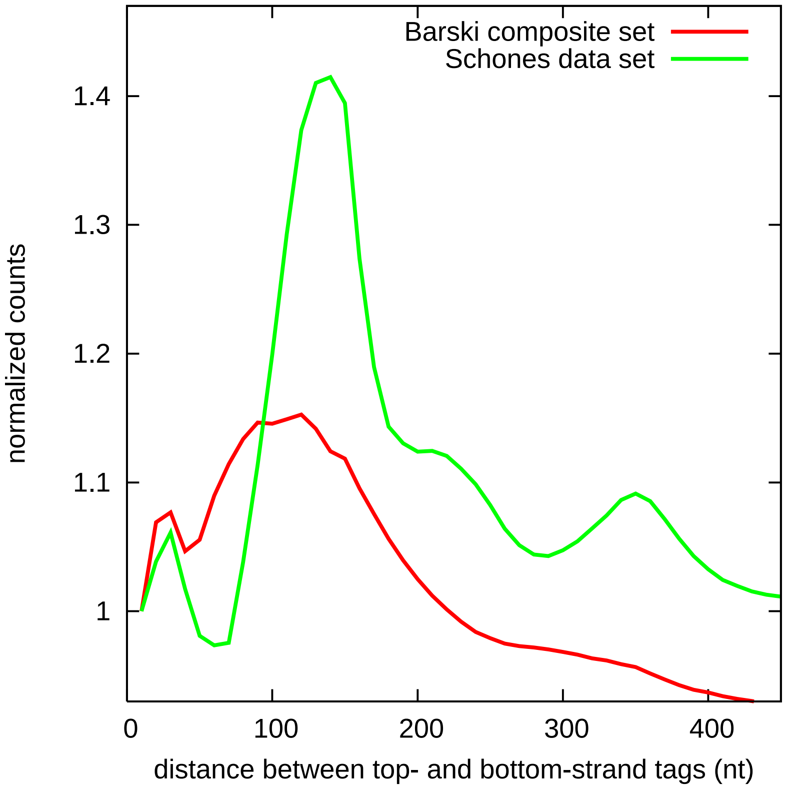

Figure S1: Histogram of distances between short-read tags mapped to the top- and bottom-strands for the composite Barski data set and the Schones data set. The histogram consists of counts in bins of width 10 nucleotides, and the y-axis is normalized by the count in the first bin.

The NPS algorithm looks for regions in which sets of plus-strand tags and minus-strand tags are separated by a distance that corresponds to a single nucleosome core. Based on the analysis shown in this figure, it is clear that the Schones data set provides a more consistent set of tags separated by a distance of approximately 140 nucleotides. The agglomeration of the 21 separate ChIP-Seq experiments in the Barski data set is less enriched for tags separated by the expected distance, and the observed spread is significantly wider. Based on this analysis, we chose to proceed with our analysis using only the Schones data set.
