# Supplementary material for: Learning a Weighted Sequence Model of the Nucleosome Core and Linker Yields More Accurate Predictions in Saccharomyces cerevisiae and Homo sapiens
Source: PLoS Comput Biol. 2010 Jul 8;6(7):e1000834. doi: 10.1371/journal.pcbi.1000834 (PMC2900294; doi:10.1371/journal.pcbi.1000834)
Supplement: Figure S2 — Single-nucleotide PSFM computed across all nucleosome sequences, using only the top-strand sequence centered at each nucleosome dyad. The bottom figure zooms in on the 300 positions centered at the dyad. The gradual increase in the total GC-content shown in Figure∼S1 as the distance to the nucleosome dyad decreases is due to a relative over-representation of nucleosome positions in the Zhang dataset in GC-rich regions of the genome. This over-representation of nucleosome positions in GC-rich regions is also described by Zhang et al. [2] and is attributed to a combination of the ChIP-selection for histone modifications that are known to be over-represented in genes and near promoters, and the known GC-bias in the coverage of Solexa sequencing. (0.10 MB PDF) [file pcbi.1000834.s004.pdf]

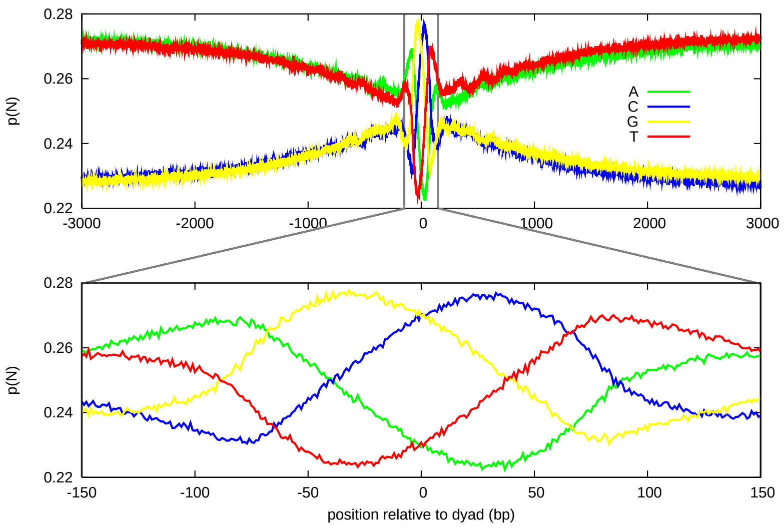

Figure S2: Single-nucleotide PSFM computed across all nucleosome sequences, using only the top-strand sequence centered at each nucleosome dyad. The bottom figure zooms in on the 300 positions centered at the dyad.

The gradual increase in the total GC-content shown in Figure S1 as the distance to the nucleosome dyad decreases is due to a relative over-representation of nucleosome positions in the Zhang dataset in GC-rich regions of the genome. This over-representation of nucleosome positions in GC-rich regions is also described by Zhang *et al.* [2] and is attributed to a combination of the ChIP-selection for histone modifications that are known to be over-represented in genes and near promoters, and the known GC-bias in the coverage of Solexa sequencing.
