# Supplementary material for: Learning a Weighted Sequence Model of the Nucleosome Core and Linker Yields More Accurate Predictions in Saccharomyces cerevisiae and Homo sapiens
Source: PLoS Comput Biol. 2010 Jul 8;6(7):e1000834. doi: 10.1371/journal.pcbi.1000834 (PMC2900294; doi:10.1371/journal.pcbi.1000834)
Supplement: Figure S3 — The x-axis represents the maximum absolute variation observed in a mono-nucleotide pattern derived by averaging 438,000 individual DNA sequences of length 151 bp each. The null-model distribution was obtained empirically from 800,000 random sets of DNA fragments. (a) shows the null model distributions based on random sampling for each of the 4 nucleotides as well as the observed deltas based on the nucleosome pattern derived from the Zhang positions, and (b) shows the null distributions at higher resolution. (0.01 MB PDF) [file pcbi.1000834.s005.pdf]

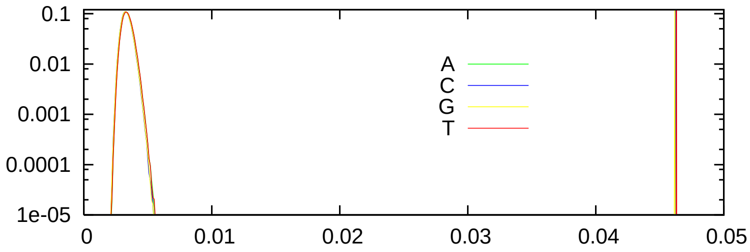

(a) Null model distributions and observed Δ's

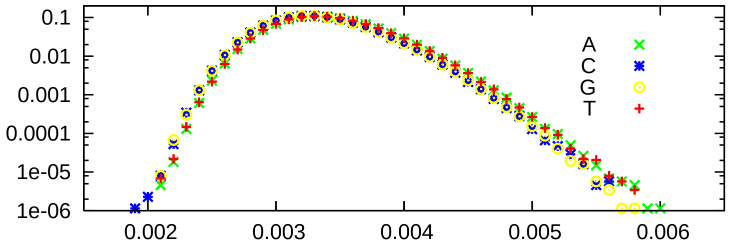

(b) Null model distributions

Figure S3: The x-axis represents the maximum absolute variation observed in a mono-nucleotide pattern derived by averaging 438,000 individual DNA sequences of length 151 bp each. The null-model distribution was obtained empirically from 800,000 random sets of DNA fragments. (a) shows the null model distributions based on random sampling for each of the 4 nucleotides as well as the observed Δ's based on the nucleosome pattern derived from the Zhang positions, and (b) shows the null distributions at higher resolution.
