# Supplementary material for: Learning a Weighted Sequence Model of the Nucleosome Core and Linker Yields More Accurate Predictions in Saccharomyces cerevisiae and Homo sapiens
Source: PLoS Comput Biol. 2010 Jul 8;6(7):e1000834. doi: 10.1371/journal.pcbi.1000834 (PMC2900294; doi:10.1371/journal.pcbi.1000834)
Supplement: Figure S4 — The nucleosome positions are over-represented in GC-rich regions, but the pattern is observed across different ranges of AT content and is strongest in the very high AT content subset (c) and weakest in the very low AT content subset (f). (a) shows the pattern derived from all of the nucleosome positions and (b) through (f) show the patterns derived from non-overlapping quintiles, divided according to AT content. (For each plot, A (green) and T (red) are plotted against the y-axis on the left, while C (blue) and G (yellow) are plotted against the y-axis on the right.) (0.04 MB PDF) [file pcbi.1000834.s006.pdf]

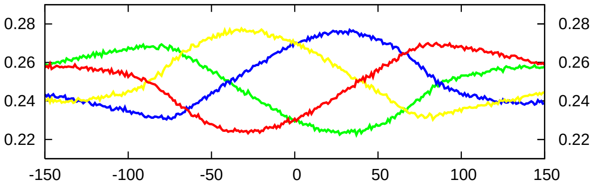

(a) pattern derived from all nucleosome sequences

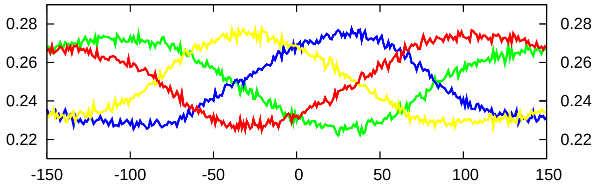

(b) middle 20% by AT content (between 0.47 and 0.51)

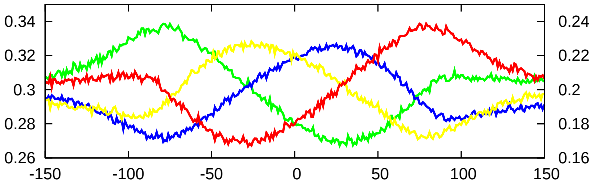

(c) very high AT content (above 0.56)

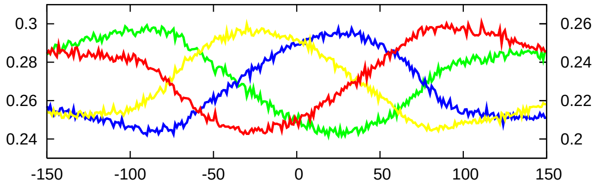

(d) high AT content (between 0.51 and 0.56)

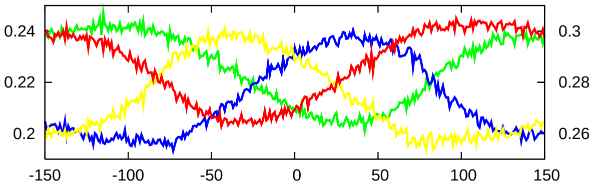

(e) low AT content (between 0.40 and 0.47)

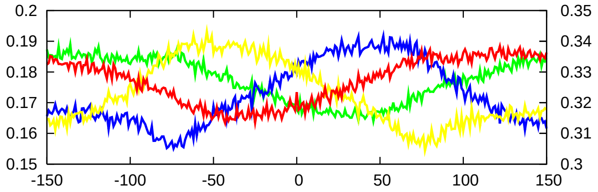

(f) very low AT content (below 0.40)

Figure S4: The nucleosome positions are over-represented in GC-rich regions, but the pattern is observed across different ranges of AT content and is strongest in the very high AT content subset (c) and weakest in the very low AT content subset (f). (a) shows the pattern derived from all of the nucleosome positions and (b) through (f) show the patterns derived from non-overlapping quintiles, divided according to AT content. (For each plot, A (green) and T (red) are plotted against the y-axis on the left, while C (blue) and G (yellow) are plotted against the y-axis on the right.)
