# Supplementary material for: Learning a Weighted Sequence Model of the Nucleosome Core and Linker Yields More Accurate Predictions in Saccharomyces cerevisiae and Homo sapiens
Source: PLoS Comput Biol. 2010 Jul 8;6(7):e1000834. doi: 10.1371/journal.pcbi.1000834 (PMC2900294; doi:10.1371/journal.pcbi.1000834)
Supplement: Figure S5 — The nucleosome pattern is largely unchanged when the dataset is partitioned according to local repeat content, although the A and T curves shift upwards relative to the C and G curves. (a) 70% of the nucleosome positions are at least 30 bases away from a repeat; (b) 19% are in a repeat that extends at least 30 bases to both sides; and (c) 11% are near the edge of a repeat. The Pearson correlation between local AT content and local repeat fraction is +0.23 (although Alu repeats are only 45–50% AT, other repeats generally have higher AT content, particularly LINE1 repeats which are approximately 65% AT). The increased noise in the patterns in (b) and (c) is due to the smaller number of sequences used to create these two patterns. (0.02 MB PDF) [file pcbi.1000834.s007.pdf]

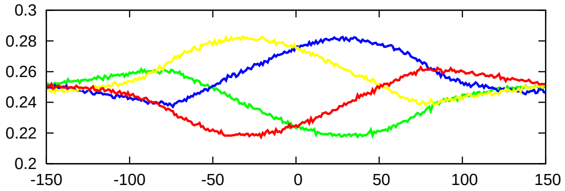

(a) at least 30bp away from a repeat region (307,873 sequences)

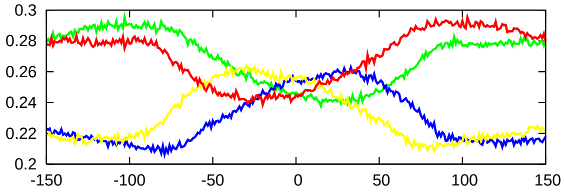

(b) at least 30bp away from a non-repeat region (81,918 sequences)

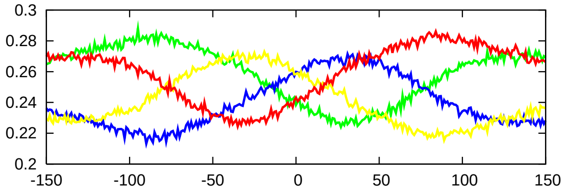

(c) within 30bp of both repeat and non-repeat regions (48,842 sequences)

Figure S5: The nucleosome pattern is largely unchanged when the dataset is partitioned according to local repeat content, although the A and T curves shift upwards relative to the C and G curves. (a) 70% of the nucleosome positions are at least 30 bases away from a repeat; (b) 19% are in a repeat that extends at least 30 bases to both sides; and (c) 11% are near the edge of a repeat. The Pearson correlation between local AT content and local repeat fraction is +0.23 (although Alu repeats are only 45-50% AT, other repeats generally have higher AT content, particularly LINE1 repeats which are approximately 65% AT). The increased noise in the patterns in (b) and (c) is due to the smaller number of sequences used to create these two patterns.
