# Supplementary material for: Learning a Weighted Sequence Model of the Nucleosome Core and Linker Yields More Accurate Predictions in Saccharomyces cerevisiae and Homo sapiens
Source: PLoS Comput Biol. 2010 Jul 8;6(7):e1000834. doi: 10.1371/journal.pcbi.1000834 (PMC2900294; doi:10.1371/journal.pcbi.1000834)
Supplement: Figure S6 — The four possible permutations of the original pattern were correlated against all nucleosome sequences. Each sequence was then assigned to one of four classes, and new patterns derived after realigning the sequences according to the peak correlation offset (green = A, red = T, blue = C, yellow = G). (0.03 MB PDF) [file pcbi.1000834.s008.pdf]

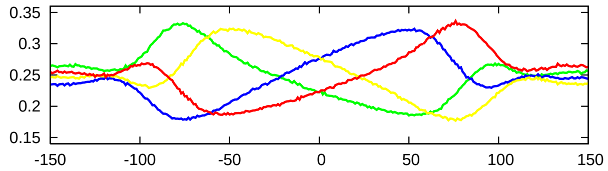

(a) pattern AGCT : 25.0% of all sequences

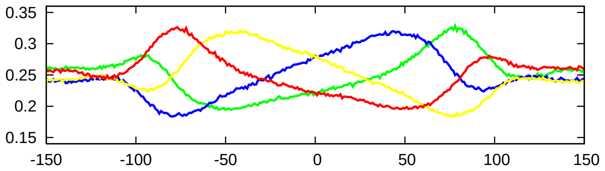

(b) pattern TGCA : 11.5% of all sequences

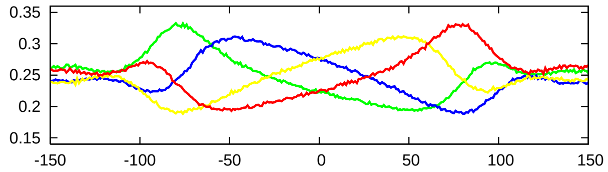

(c) pattern ACGT : 11.5% of all sequences

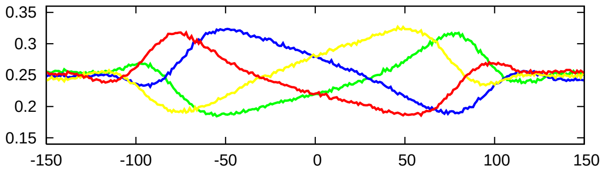

(d) pattern TCGA : 8.5% of all sequences

Figure S6: The four possible permutations of the original pattern were correlated against all nucleosome sequences. Each sequence was then assigned to one of four classes, and new patterns derived after realigning the sequences according to the peak correlation offset (green=A, red=T, blue=C, yellow=G).

We refer to the dominant pattern illustrated in Figure S1 as AGCT, based on the 5’ to 3’ ordering of the local maxima. Just as the pattern is its own reverse complement, so is this acronym. There are three other possible 4-nucleotide sequences that obey this rule and also follow the common nucleosome model—higher AT content in the linker regions, and higher GC content in the core: ACGT, TGCA, and TCGA. We hypothesized that one or more of these alternative patterns may be present within a subset of the nucleosomal sequences but not visible in the overall average. In order to test this hypothesis, we modeled all four pattern variants and partitioned the input set of sequences according to which of the four patterns best matched each individual sequence (if the input sequence did not correlate well with any pattern, it was left out). New patterns were then reconstructed using these non-overlapping subsets of sequences. The four reconstructed patterns are shown in Figure S6a-d, labeled with the fraction of the total set of input sequences used to create each one. As expected, the dominant pattern (AGCT) was the best match for more sequences than any other (25%), while the next two patterns(TGCA and ACGT) appear to exist in comparable proportions within this dataset. The fourth pattern (TCGA) appears to occur much less frequently. Together these four reconstructed patterns represent 56.5% of the nucleosomal sequences in this dataset.
