# Supplementary material for: Learning a Weighted Sequence Model of the Nucleosome Core and Linker Yields More Accurate Predictions in Saccharomyces cerevisiae and Homo sapiens
Source: PLoS Comput Biol. 2010 Jul 8;6(7):e1000834. doi: 10.1371/journal.pcbi.1000834 (PMC2900294; doi:10.1371/journal.pcbi.1000834)
Supplement: Figure S7 — A subset of the nucleosome patterns derived from the training set of ∼200,000 nucleosome dyad positions. The pattern for T is a reflection of the A pattern across the (vertical) dyad axis, and similarly the TT pattern is a mirror image of the AA pattern. (The same holds for C/G and CC/GG.) The patterns for dinucleotides which are their own reverse complements are symmetrical about the dyad axis. Intriguingly, the normalized patterns for CG and GC are nearly identical to one another, suggesting that these dinucleotides play the same role in positioning nucleosomes despite their dramatically different occurrence rates. (0.02 MB PDF) [file pcbi.1000834.s009.pdf]

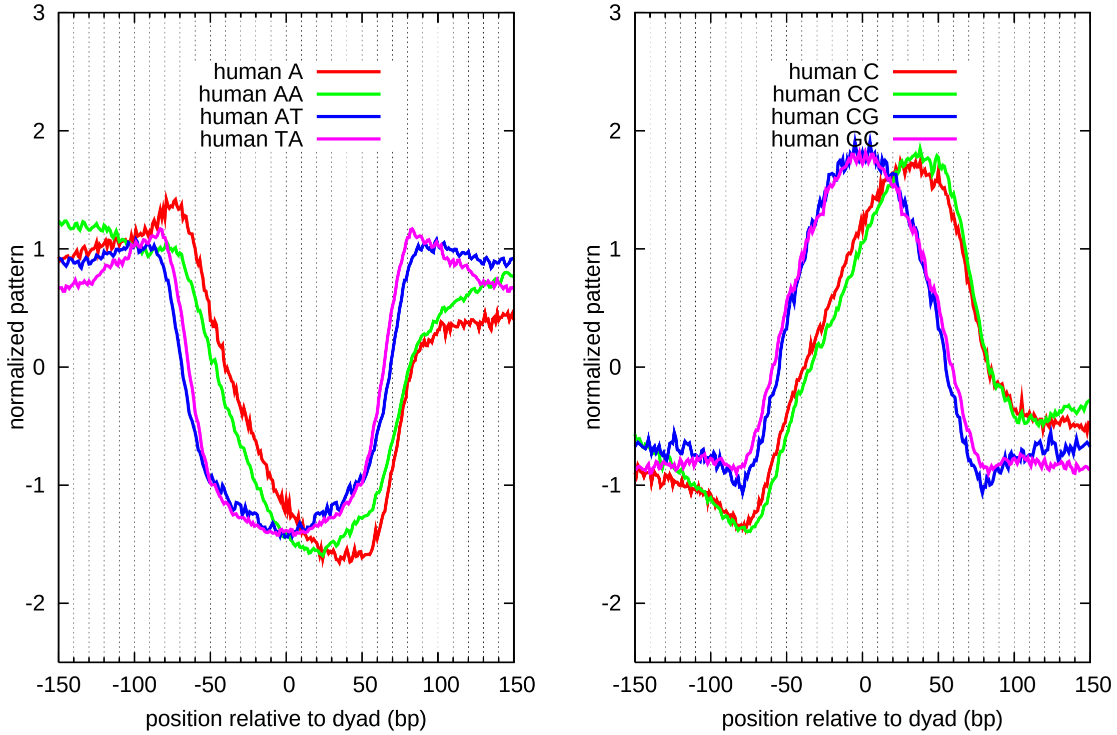

Figure S7: A subset of the nucleosome patterns derived from the training set of ∼200,000 nucleosome dyad positions. The pattern for T is a reflection of the A pattern across the (vertical) dyad axis, and similarly the TT pattern is a mirror image of the AA pattern. (The same holds for C/G and CC/GG.) The patterns for dinucleotides which are their own reverse complements are symmetrical about the dyad axis. Intriguingly, the normalized patterns for CG and GC are nearly identical to one another, suggesting that these dinucleotides play the same role in positioning nucleosomes despite their dramatically different occurrence rates.
