# Supplementary material for: Learning a Weighted Sequence Model of the Nucleosome Core and Linker Yields More Accurate Predictions in Saccharomyces cerevisiae and Homo sapiens
Source: PLoS Comput Biol. 2010 Jul 8;6(7):e1000834. doi: 10.1371/journal.pcbi.1000834 (PMC2900294; doi:10.1371/journal.pcbi.1000834)
Supplement: Figure S8 — These figures show the S. cerevisiae and H. sapiens nucleosome patterns for A, AA, and AAA. Note that these S. cerevisiae patterns still contain the MNase artifact, which was most apparent in the A pattern. The Pearson correlation coefficients for each S. cerevisiae pattern and the corresponding H. sapiens pattern are: A:0.77, AA:0.88, and AAA:0.91, suggesting that the underlying oscillation common to both patterns plays a role in positioning nucleosomes across species. (0.02 MB PDF) [file pcbi.1000834.s010.pdf]

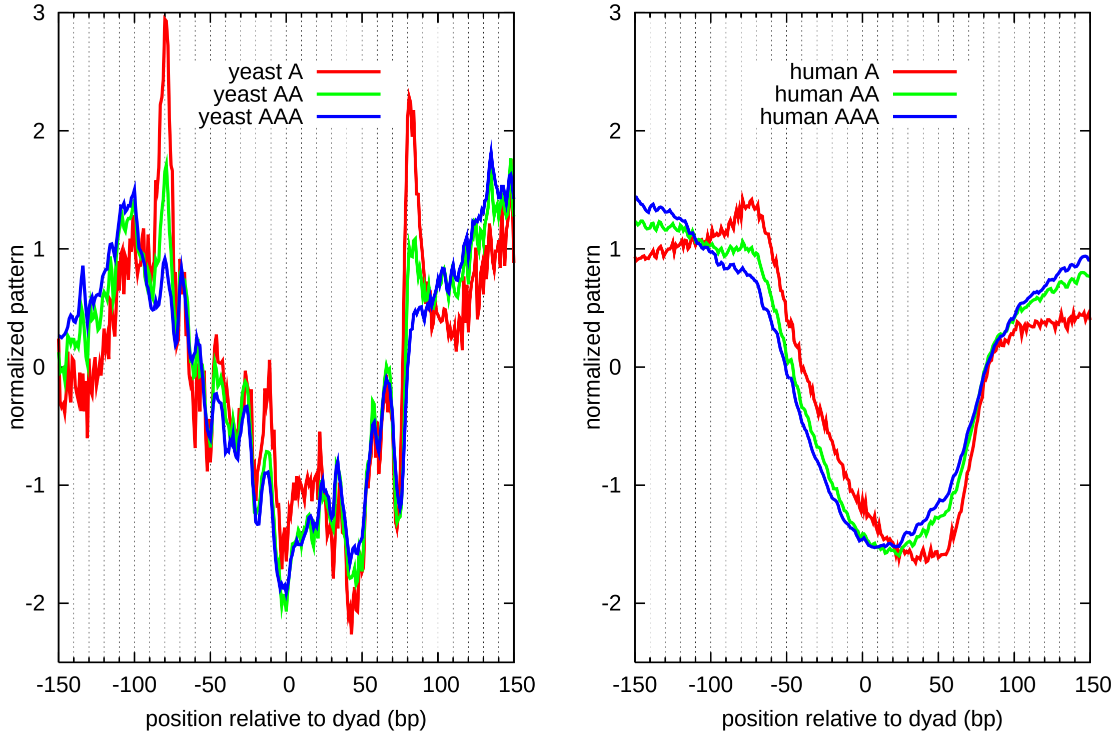

Figure S8: These figures show the yeast and human nucleosome patterns for A, AA, and AAA. Note that these yeast patterns still contain the MNase artifact, which was most apparent in the A pattern. The Pearson correlation coefficients for each yeast pattern and the corresponding human pattern are: A:0.77, AA:0.88, and AAA:0.91, suggesting that the underlying oscillation which these patterns have in common plays a role in positioning nucleosomes across species.
