# Supplementary material for: Learning a Weighted Sequence Model of the Nucleosome Core and Linker Yields More Accurate Predictions in Saccharomyces cerevisiae and Homo sapiens
Source: PLoS Comput Biol. 2010 Jul 8;6(7):e1000834. doi: 10.1371/journal.pcbi.1000834 (PMC2900294; doi:10.1371/journal.pcbi.1000834)
Supplement: Figure S9 — Area under the ROC curve as a function of the distance L between the dyad positions and the non-dyad positions on either side. Near the dyad, discrimination is difficult and the performance is not much better than random, especially in H. sapiens. As the distance from the dyad increases, performance improves, reaching a maximum in the linker, after which the performance degrades again. (0.01 MB PDF) [file pcbi.1000834.s011.pdf]

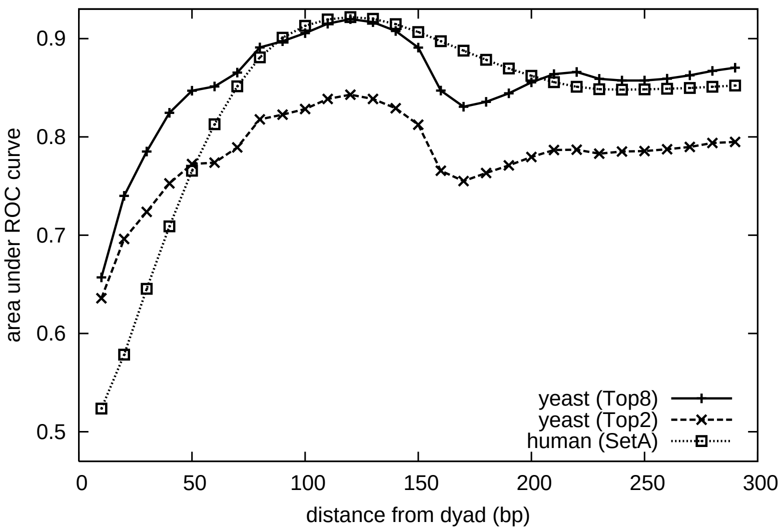

Figure S9: Area under the ROC curve as a function of the distance L between the dyad positions and the *non*-dyad positions on either side. Near the dyad, discrimination is difficult and the performance is not much better than random, especially in human. As the distance from the dyad increases, performance improves, reaching a maximum in the linker, after which the performance degrades again.
