# Supplementary material for: Learning a Weighted Sequence Model of the Nucleosome Core and Linker Yields More Accurate Predictions in Saccharomyces cerevisiae and Homo sapiens
Source: PLoS Comput Biol. 2010 Jul 8;6(7):e1000834. doi: 10.1371/journal.pcbi.1000834 (PMC2900294; doi:10.1371/journal.pcbi.1000834)
Supplement: Figure S10 — These four traces represent the average dyad score across four subsets of S. cerevisiae transcription start sites, clustered according to promoter nucleosome profile by Lee et al. [18]. The grey curves accompanying each of the dyad score traces were taken from Figure 4a in the same paper by Lee et al. (Note that the grey traces are not plotted on the same y-axis scale as the average dyad score traces, but are plotted on the same scale relative to each other.) The GO Slim biological process term most overrepresented by genes in each cluster, as reported by Lee et al. were: “response to stress” (top, red); “translation” (next, green); “ribosome biogenesis and assembly” (next, dark blue); and “organelle organization and biogenesis” (bottom, light blue). (0.02 MB PDF) [file pcbi.1000834.s012.pdf]

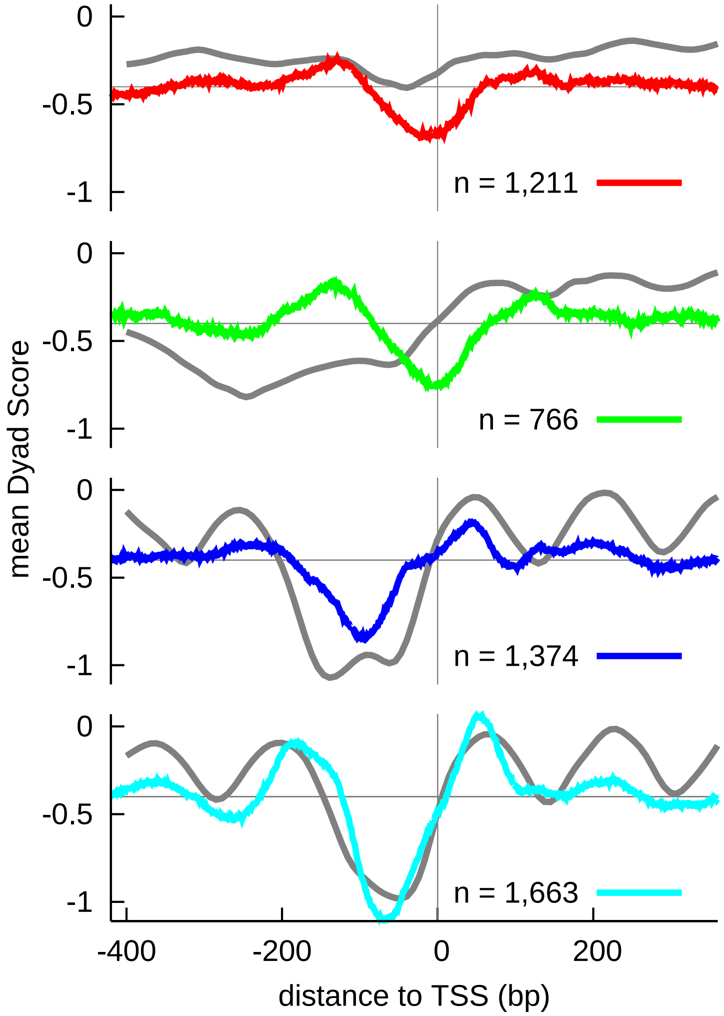

Figure S10: These four traces represent the average dyad score across four subsets of yeast transcription start sites, clustered according to promoter nucleosome profile by Lee *et al.* [17]. The grey curves accompanying each of the dyad score traces were taken from Figure 4a in the same paper by Lee *et al.* (Note that the grey traces are not plotted on the same y-axis scale as the average dyad score traces, but are plotted on the same scale relative to each other.) The GO Slim biological process term most overrepresented by genes in each cluster, as reported by Lee *et al.* were: “response to stress” (top, red); “translation” (next, green); “ribosome biogenesis and assembly” (next, dark blue); and “organelle organization and biogenesis” (bottom, light blue).
