# Supplementary material for: Learning a Weighted Sequence Model of the Nucleosome Core and Linker Yields More Accurate Predictions in Saccharomyces cerevisiae and Homo sapiens
Source: PLoS Comput Biol. 2010 Jul 8;6(7):e1000834. doi: 10.1371/journal.pcbi.1000834 (PMC2900294; doi:10.1371/journal.pcbi.1000834)
Supplement: Figure S11 — (A) Model predictions averaged over 32,000+ transcription start sites from DBTSS (red), and experimentally derived dyad curve based on the Zhang set of nucleosome positions (grey). Note the ∼40bp offset between the red and grey curves, and the prediction of a nucleosome near −100 bp, in the apparent “nucleosome free region’ (NFR) upstream of the TSS. The apparent disagreement between the predictions and the experimental averages are due to two effects: first, the experimental data only spans approximately one third of the entire set of 32,000 TSSs; and second, the locally optimal dyad score for each +1 nucleosome (for example) may vary considerably, thereby contributing unequally to the average profile. In order to verify that the ∼40 bp offset did not represent a systematic bias in our method, we created subsets of the DBTSS positions according to the relative position of an experimentally-determined dyad. We then computed the mean predicted dyad score across these subsets. Three representative subsets are shown in (B): the number of TSS in each subset is indicated by the number in the parentheses, and the mid-point of the 30 bp window is given by the number preceding the parentheses. For example, the red curve represents an average over 1252 predictions, for transcription start sites with experimental dyad positions mapped between 20 and 50 bp downstream of the TSS. The position of the peak in each of these average curves matches the mid-point of the 30 bp window almost exactly. (The grey curve in (A) is not plotted on the same y-axis as the red curve and is shown only for reference.) (0.02 MB PDF) [file pcbi.1000834.s013.pdf]

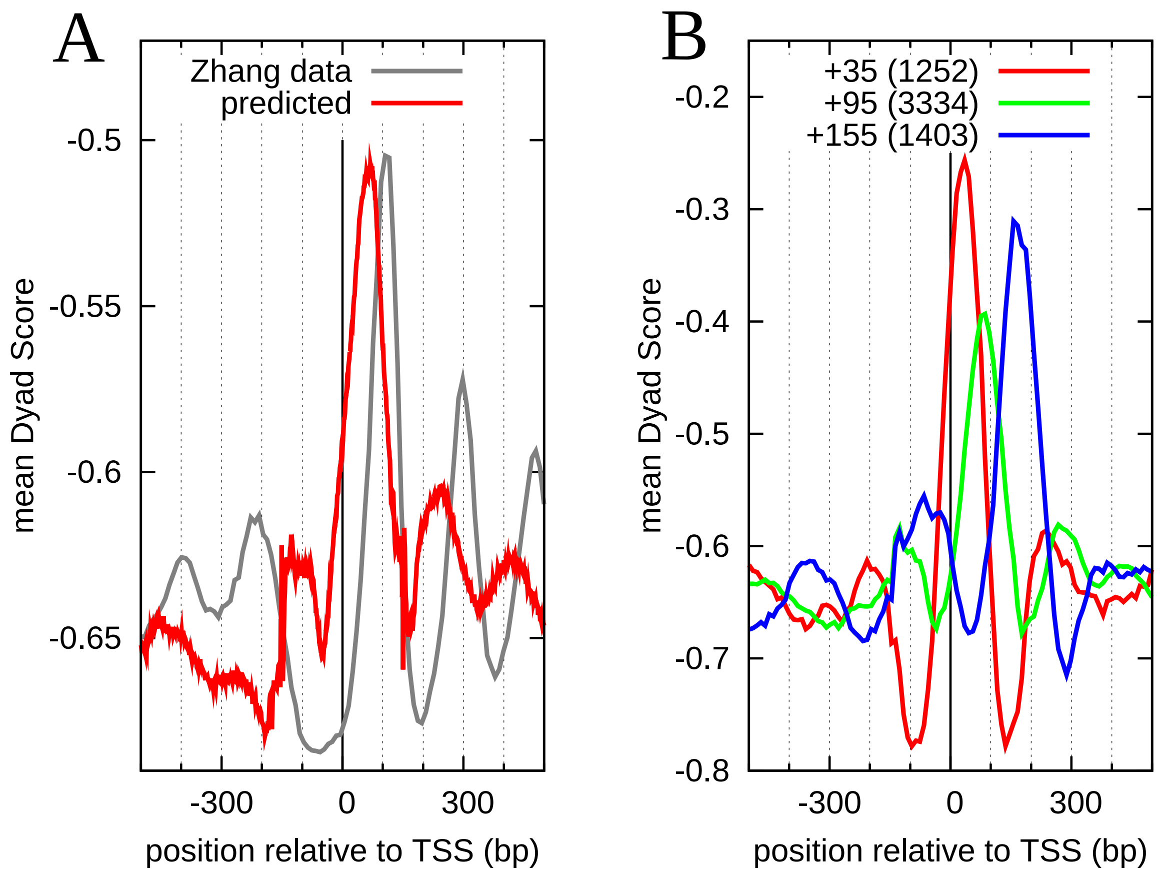

Figure S11: (A) Model predictions averaged over 32,000+ transcription start sites from DBTSS (red), and experimentally derived dyad curve based on the Zhang set of nucleosome positions (grey). Note the $\sim$40bp offset between the red and grey curves, and the prediction of a nucleosome near –100 bp, in the apparent "nucleosome free region" (NFR) upstream of the TSS. The apparent disagreement between the predictions and the experimental averages are due to two effects: first, the experimental data only spans approximately one third of the entire set of 32,000 TSSs; and second, the locally optimal dyad score for each +1 nucleosome (for example) may vary considerably, thereby contributing unequally to the average profile. In order to verify that the $\sim$40 bp offset did not represent a systematic bias in our method, we created subsets of the DBTSS positions according to the relative position of an experimentally-determined dyad. We then computed the mean predicted dyad score across these subsets. Three representative subsets are shown in (B): the number of TSS in each subset is indicated by the number in the parentheses, and the mid-point of the 30 bp window is given by the number preceding the parentheses. For example, the red curve represents an average over 1252 predictions, for transcription start sites with experimental dyad positions mapped between 20 and 50 bp downstream of the TSS. The position of the peak in each of these average curves matches the mid-point of the 30 bp window almost exactly. (The grey curve in (A) is not plotted on the same y-axis as the red curve and is shown only for reference.)
