# Supplementary material for: Learning a Weighted Sequence Model of the Nucleosome Core and Linker Yields More Accurate Predictions in Saccharomyces cerevisiae and Homo sapiens
Source: PLoS Comput Biol. 2010 Jul 8;6(7):e1000834. doi: 10.1371/journal.pcbi.1000834 (PMC2900294; doi:10.1371/journal.pcbi.1000834)
Supplement: Figure S12 — Model predictions averaged over two sets of CTCF binding sites: occupied (red) and unoccupied (green). The solid grey trace represents the experimentally-determined nucleosome occupancy derived from Figure 5B in Cuddapah et al. [42] near unbound CTCF binding sites, and the thin grey line with triangles is the nucleosome occupancy curve surrounding bound CTCF sites from Figure 3A in Zhang et al. [2]. Without CTCF binding, the sequence-dependent nucleosome-positioning does not result in a strong coherent pattern of flanking nucleosomes, in agreement with the data from Cuddapah et al. In contrast, CTCF binding results in a significantly more coherent pattern of flanking nucleosomes. The difference between the red and the green curves suggests that the set of “unoccupied” CTCF sites includes a number of false-positives which appear to have significantly different flanking sequence characteristics. (The grey curves are not plotted on the same y-axis as the red and green curves and are shown only for reference.) (0.02 MB PDF) [file pcbi.1000834.s014.pdf]

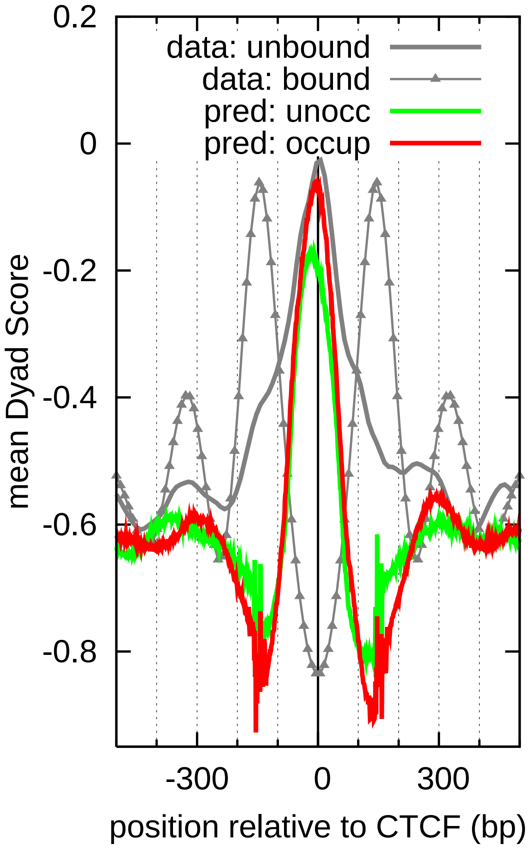

Figure S12: Model predictions averaged over two sets of CTCF binding sites: occupied (red) and unoccupied (green). The solid grey trace represents the experimentally-determined nucleosome occupancy derived from Figure 5B in Cuddapah *et al.* [41] near unbound CTCF binding sites, and the thin grey line with triangles is the nucleosome occupancy curve surrounding bound CTCF sites from Figure 3A in Zhang *et al.* [2]. Without CTCF binding, the sequence-dependent nucleosome-positioning does not result in a strong coherent pattern of flanking nucleosomes, in agreement with the data from Cuddapah *et al.*. In contrast, CTCF binding results in a significantly more coherent pattern of flanking nucleosomes. The difference between the red and the green curves suggests that the set of "unoccupied" CTCF sites includes a number of false-positives which appear to have significantly different flanking sequence characteristics. (The grey curves are not plotted on the same y-axis as the red and green curves and are shown only for reference.)
